# *Paris* spp (Liliaceae): a review of its botany, ethnopharmacology, phytochemistry, pharmacological activities, and practical applications

**DOI:** 10.3389/fphar.2025.1570818

**Published:** 2025-05-30

**Authors:** Ji Zhou, Binbin Liao, Julian Miao, Xubing Chen

**Affiliations:** ^1^ College of Pharmaceutical Science, Dali University, Dali, China; ^2^ College of Pharmaceutical Science, Hubei University of Chinese Medicine, Hubei, China

**Keywords:** *Paris* L., steroidal saponins, ethnopharmacology, phytochemistry, pharmacological activities, applications

## Abstract

Paris spp., as a traditional medicinal plant, are widely used globally due to their diverse therapeutic properties, including clearing heat and detoxifying, reducing swelling and relieving pain, calming the liver and suppressing convulsions. This review summarizes the research progress of *Paris* species in the fields of ethnopharmacology, phytochemistry, pharmacological effects, and applications in recent years. The study systematically retrieved information related to the keywords “phytochemistry,” “pharmacology,” and “toxicology” from authoritative databases such as CNKI, PubMed, Elsevier, Web of Science, and SpringerLink, using “*Paris* L.” as a keyword to collect research materials related to this genus. During this process, a total of 431 metabolites were isolated and identified, with steroidal saponins being the most abundant. In addition to their medicinal uses, *Paris* spp. have also been applied in hair care products, cosmetics, and health products. Despite their demonstrated significant pharmacological activities and potential clinical applications, the field of *Paris* spp. research still faces several challenges. For example, the specific mechanisms of action against certain diseases are not fully understood, and multiple studies have shown that *Paris* species’ extracts may cause adverse reactions and even toxicity. Therefore, further in-depth research and systematic evaluation are needed to promote the safe application and clinical translation of *Paris* spp.

## 1 Introduction

Approximately 70% of the global population relies on botanical drug, which has great potential in global healthcare due to its diverse preparation methods and natural safety. However, further development is needed to address cost and market acceptance issues (Che et al., 2013; Shen et al., 2021). *Paris* spp. belonging to the Liliaceae family, consists of perennial botanical drug classified as rare and endangered plants in central China. After years of cultivation and evolution, this botanical drug has been widely distributed, such as India, China, Bhutan, Laos, Myanmar, Nepal, Thailand, and Vietnam (Li et al., 2024). They are mainly distributed in southwestern China, such as Yunnan, Guizhou, Sichuan, and other places. *Paris* spp. are traditional and commonly used Chinese medicinal materials (Meng et al., 2020). The rhizome is used in medicine with a bitter taste and slightly cold nature; it is slightly toxic and associated with the liver meridian. It has the effects of clearing heat and detoxifying, reducing swelling and relieving pain, and calming the liver to alleviate convulsions (Wang et al., 2022; Xiao et al., 2016; Zhang et al., 2015). In addition, it is also used to treat trauma, abscesses, mumps, mastitis, etc., and can also serve as an antidote for snake bites (Nie et al., 2022).

Modern research indicates that members of the genus *Paris* is rich in steroidal saponins, triterpenoids, polysaccharides, flavonoids, and other metabolites. Experiments have found that these metabolites possess pharmacological activities such as anticancer (He et al., 2015), anti-tumor (Yao et al., 2017), antioxidant (He et al., 2023), and antibacterial effects (Sun et al., 2023). Due to their wide range of pharmacological actions, they are used to treat various diseases in internal medicine, gynecology, otorhinolaryngology, orthopedics, and other fields (Gu et al., 2023). Although *Paris* species has a wide range of pharmacological effects, research has shown that high doses of the *Paris* spp*.* can cause toxic reactions to the reproductive, nervous, and digestive systems (Chen and Yan, 2012; Yang et al., 2018). *Paris* species holds extremely high economic value in medicinal use, but it should be handled cautiously to ensure its pharmacological effects are exerted safely and effectively.

*Paris* spp. is an important medicinal plant, serving as one of the raw materials for more than 80 traditional Chinese patent medicines, such as “Yunnan Red Medicine Powder” and “Gong Xue Ning Capsules” (Zhang et al., 2024). Members of the genus *Paris* has a high medicinal value, with applications ranging from wound healing to cancer treatment. The increasing demand has led to a rise in market prices, pushing the *Paris* species in China to extinction (Thakur et al., 2023).

Through an in-depth review of the literature, this paper comprehensively collates the medicinal history and current status of *Paris* species used by ethnic minorities in China and various countries, providing significant references for the conservation and sustainable utilization of wild resources. Given the endangered status of *Paris* species, the search for alternative resources has become an urgent priority. Despite the notable pharmacological activities of *Paris* species against multiple diseases, some mechanisms of action remain unclear. Additionally, their extracts may cause adverse reactions and even toxicity, which to some extent, restricts their widespread clinical application. Therefore, there is an urgent need for further in-depth research on their chemical constituents, pharmacological effects, and toxic side effects, as well as the establishment of corresponding quality control standards and risk assessment systems, to facilitate the successful translation of *Paris* species from basic research to clinical practice. This paper systematically reviews the research progress of *Paris* spp. in the fields of ethnopharmacology, botany, phytochemistry, pharmacology, and toxicology, offering comprehensive information and suggestions for clinical application and future research.

## 2 Botanical research

### 2.1 Geographical distribution

**FIGURE 1:**
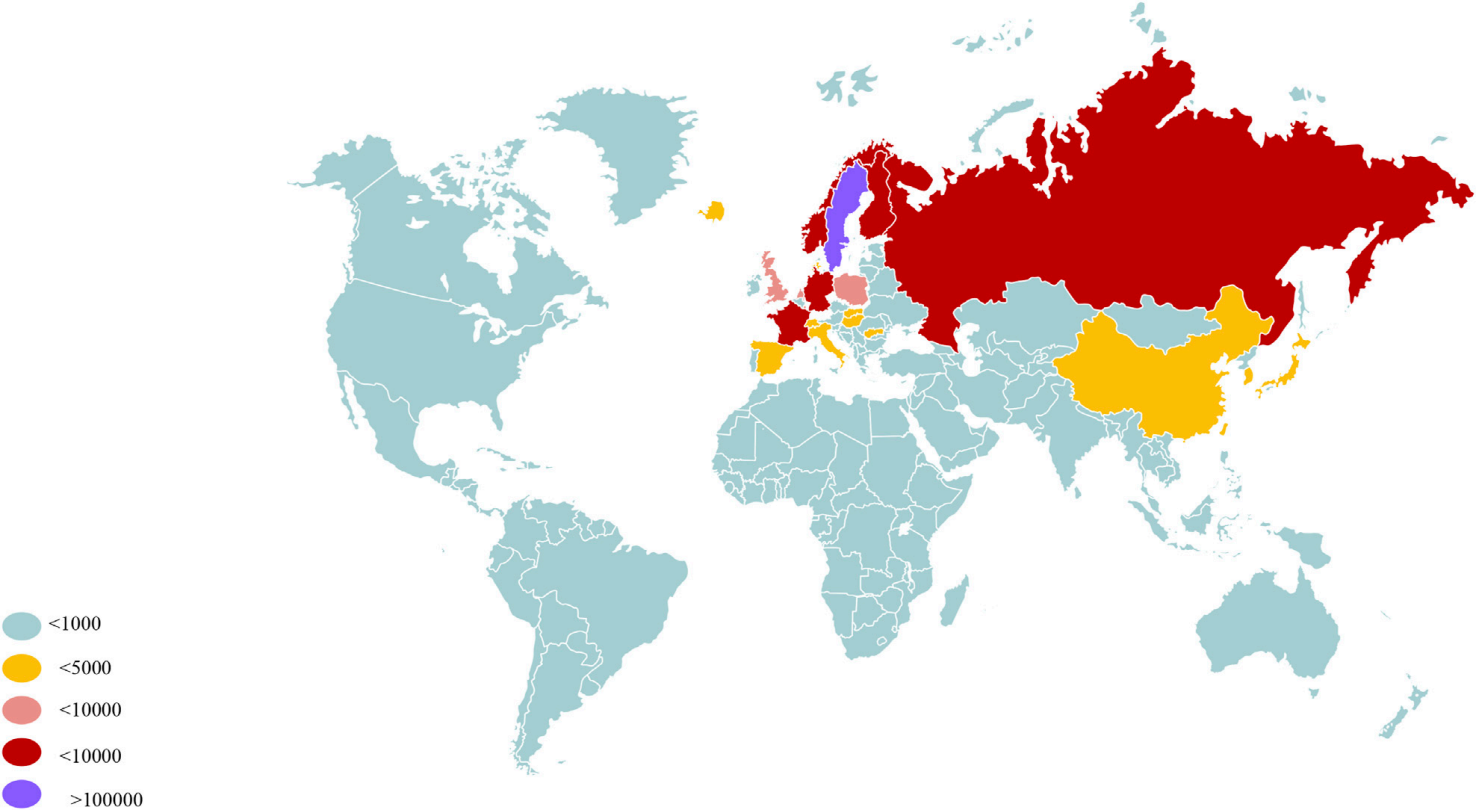
Global distribution of *Paris* spp.

*Paris* spp. belonging to the Liliaceae or Melanthiaceae family, and its specific classification varies in different studies. Due to differences in temperature, humidity, and species, *Paris* spp. widely thrives in the temperate and subtropical regions of Europe and northern Asia (Ding et al., 2021). Organizing the global distribution of medicinal plants helps to assess the total amount of resources and their potential value for use. It provides a basis for the conservation of rare plants, identifies areas where resources are concentrated for rational harvesting, and prevents depletion. At the same time, studying their distribution patterns can reveal the latitudinal gradient characteristics of biodiversity, protect genetic diversity, and provide genetic resources to cope with environmental changes. In addition, organizing distribution information can also provide clues for discovering new active ingredients, study the geographical variations of these ingredients, and combine traditional medicine with modern pharmacology to facilitate the development of new drugs. According to GBIF (https://www.gbif.org), a global distribution map of *Paris* spp. Figure 1 has been drawn.

### 2.2 Botanical characteristics

**FIGURE 2:**
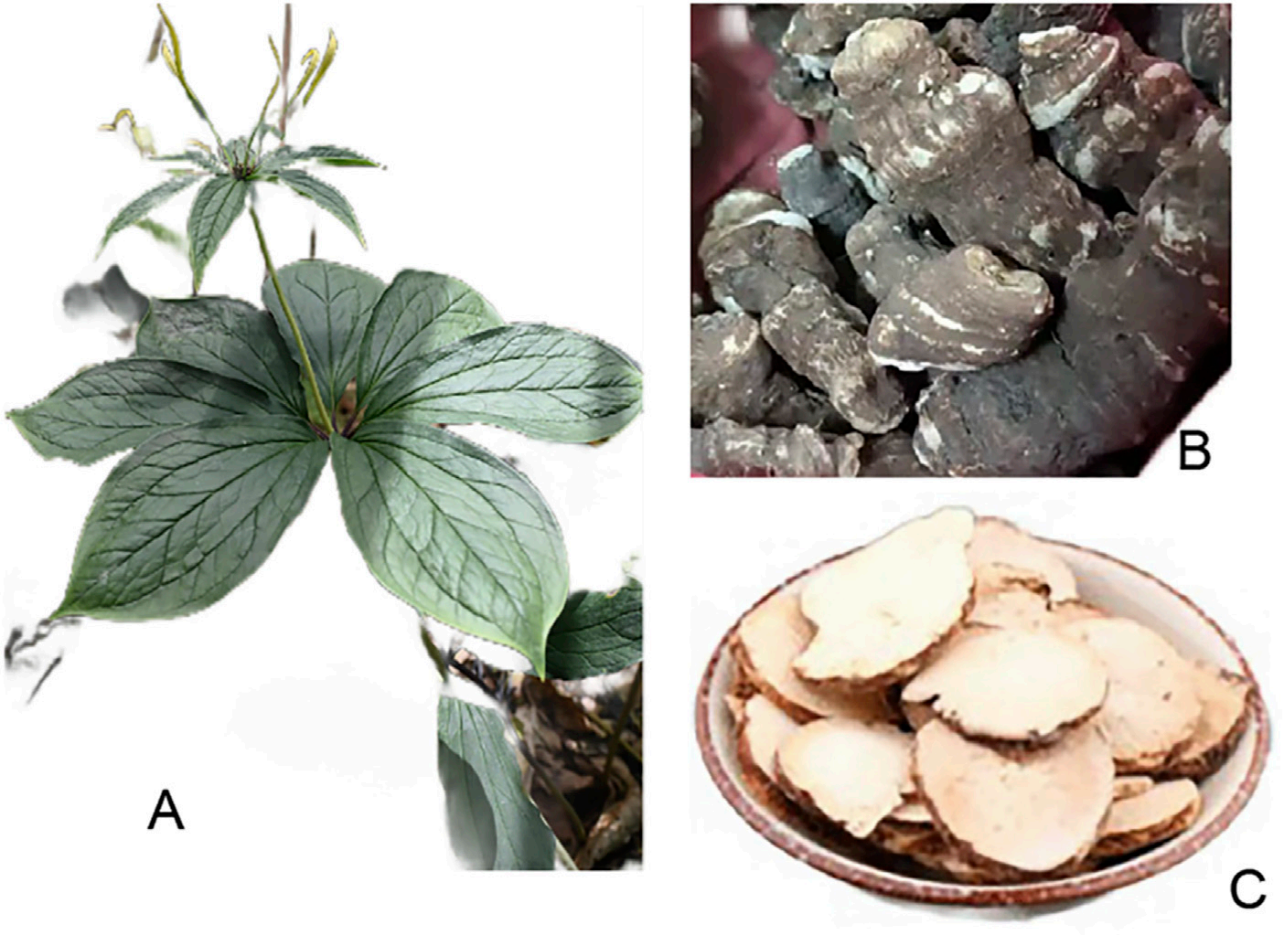
The aerial tissues **(A)**, medicinal root tissues **(B)**, and commercial presentation **(C)** of *Paris* spp.

Members of the genus *Paris* is known for its distinctive botanical features and is valued for its medicinal properties. *Pari*s spp. belongs to the family Liliaceae, is a perennial herbaceous plant distributed in the temperate and subtropical regions of the Eurasian continent. According to Flora of China (1978), the botanical characteristics of the *Paris* species are described below: (1) Rhizome: Fleshy, cylindrical, and varying in thickness with nodes. (2) Stem: Erect and unbranched, with one to three membranous sheaths at the base. (3) Leaves: Usually four or more, whorled at the top of the stem, with three central veins and a reticulate network of minor veins. (4) Flowers: Solitary in the center of the whorl, with separate, persistent perianth parts arranged in two whorls. The outer whorl is leaf-like, while the inner whorl is filamentous. Stamens are in the same number as the perianth parts, with slender, flat filaments and linear or short, basifixed anthers. (5) Ovary: Nearly spherical or conical, 4–10 locular, with a discoid stylar base or none, and a short or slender style with 4–10 branches. (6) Fruit: A capsule or berry-like capsule, smooth or angled, containing more than ten to several dozen seeds. (7) Seedlings: Germinated from seeds or propagated from rhizome pieces, with one heart-shaped leaf. (8) Germination: Generally has a low rate. (9) Medicinal Value: The genus has unique morphological characteristics and high medicinal value, with rhizomes used for medicinal purposes, particularly for snakebites, contusions, and unexplained swellings. Figure 2 displays the botanical Characteristics of *Paris* species.

## 3 Traditional uses

The *Paris* species is distributed in many regions of the world, such as India, China, Vietnam, and Germany (Li et al., 2012). *Paris* spp. not only has traditional applications among the ethnic minorities in China, but also have extensive pharmacological uses in many countries. But the traditional uses of *Paris* species vary significantly across different countries worldwide. For example, in Nepal, it is used for treating gastric pain,menstrual pain. In India, its rhizome is not only used for reducing fever, but it can also be made into a paste-like food. In Thailand, the rhizome is used for reducing fever, promoting wound healing, and relaxing muscles. These differences are not only reflected in the plant parts used and their therapeutic effects but also closely related to the medical traditions and cultural backgrounds of each country. This diversity provides rich material for cross-cultural ethnopharmacological research and lays the foundation for future exploration of its chemical constituents and pharmacological actions, as well as the development of targeted drugs. The specific information on the traditional uses of *Paris* spp. in different countries is shown in Table 1 below.

**TABLE 1:** Traditional uses of *Paris* L. in different countries.

Country	Vernacular names	Plant parts	Ethnomedicinal uses	References
Nepal	Bako	Rhizome	Gastric pain,menstrual pain, dressing wounds and removing worms	Malla et al. (2015)
China	Chong lou	Rhizome	Dysfunctional uterine bleeding, neurodermatitis, surgical infammation and cancer	Cheng et al. (2024)
India	Dudhibauj	Rhizome	Fever, pasty edible, antidote for snake and insect venom	Sharma et al. (2015)
Vietnam	Trong lâu nhiêu lá	Rhizome	Injury	Panyadee et al. (2024)
Thailand	—	Rhizome	Reducing fever, healing wounds and relaxing muscles	Ye et al. (2025)
Korea	—	Rhizome	Treating asthma, furuncles and chronic bronchitis	Ye et al. (2025)
UK	Daiswa Paris	Rhizome	Wounds, induce vomiting, as an antidote for arsenic and mercur poisoning	Ye et al. (2025)

## 4 Medicinal use by ethnic minorities

People have accumulated a wealth of traditional medicinal knowledge through their long-term struggle against diseases, with China’s traditional medicine, known for its uniqueness, becoming an essential part of the world’s traditional medicine. With its rich biodiversity and cultural diversity, Yunnan Province has become a frontier province with many ethnic minorities’ traditional medicine theories and practical experiences (Yu, 2006; Zhu, 2023).

*Paris* spp., also known as Zaoxiu, was first recorded in “Shennong Bencao Jing”: “Bitter in taste and slightly cold in nature, primarily treats conditions like epilepsy with symptoms such as convulsions and tongue protrusion, heat sensations in the abdomen, mania, vaginal ulcers, expels three types of parasites, and detoxifies snake venom” (Zhang et al., 2023a). Members of the genus *Paris* possesses the effects of clearing heat and detoxifying, reducing swelling and relieving pain, cooling the liver, and calming convulsions. It is used to treat sore throat and swelling boils and carbuncles, pain from falls and impacts (Guo et al., 2018), bites from snakes and insects (Wang et al., 2016), and convulsions due to fright (Liu et al., 2019). It is essential in traditional Chinese patent medicines such as “Yunnan Baiyao” and “Gong Xue Ning.”

Ethnic minorities use *Paris* species to treat diseases, similar to the records in the “Pharmacopoeia of the People’s Republic of China” (2020 edition).It is mainly used to treat abscesses, carbuncles, bites from snakes and insects, pain from falls and impacts, and convulsions due to fright. In the process of using *Paris* spp. to treat related diseases, ethnic minorities have shown treatments for some particular diseases, such as the Bai people using it to treat neuralgia and digestive system cancers (Jiang, 2017); the Dai people using it for submandibular lymphadenitis; the She people using it for diphtheria and Japanese encephalitis; the Tujia people using it for heart disease; the Yao people for stomach pain and boils; the Zhuang people for lymph node tuberculosis and mumps; and the Dong people for convulsions due to fright, among others.

Due to long-term over-exploitation through predatory harvesting, the resources of *Paris* spp. have suffered devastating damage and are on the verge of depletion, which are faced with a severe shortage, unable to meet production needs. Seeking new alternative resources from ethnic or folk medicines is a primary way to develop new sources of drugs (Pei, 2007). Based on relevant literature review and analysis, this paper has analyzed the primary diseases treated with *Paris* spp. For the protection and development of *Paris* spp., it is essential to formulate conservation strategies. These strategies should include the establishment of nature reserves, the initiation of research on artificial propagation and cultivation, and the search for alternative resources to reduce dependence on this plant. In ethnic minority medicine, intending to provide a reference for developing and utilizing resources of *Paris s*peci*es.* The medicinal use classified by ethnic minorities is detailed in Table 2.

**TABLE 2:** medicinal use by ethnic minorities.

Ethnicity	Traditional applications	References
Yao	Sore throat and swelling, childhood convulsions, snake bites, carbuncles and toxic swellings, furuncles, mumps, bruises and sprains	Li et al. (2005), Ma and Ye (2013), Medica et al. (2009), Wang et al. (2018)
Miao	Crbuncles and toxic swellings, throat swelling and pharyngitis, mastitis, snake and insect bites, bruises and sprains, convulsions, and tremors	Zhang and Zou (2017)
Shui	Clearing heat and detoxifying, reducing swelling and relieving pain, cooling the liver, and settling convulsions	Zhao and Yang (2017)
Bai	Carbuncles, scrofula, mastitis, bronchitis, lymph node tuberculosis, stomach pain, neuralgia, internal and external bleeding, bruises and sprains, fractures, improving appetite and digestion, expelling parasites, digestive system cancers, abdominal spasmodic pain, insect bites	Jiang (2017), Jiang and Xiao (2021), Ma and Ye (2013)
Hakka	Abscesses, tuberculosis of the lungs with chronic cough, bruises and sprains, red and swollen carbuncles and toxic conditions, snake and insect bites, lymph node tuberculosis, osteomyelitis, and other symptoms	Cheng and Chen (2016)
Li	Snake bites, stomach pain, unexplained swelling, toxic conditions, mumps, carbuncles, and boils	Dai and Mei (2008)
Yi	Breaking down and eliminating accumulations, reducing swelling and dispersing blood stasis, promoting blood circulation and relieving pain, gynecological cancers, bruises, and sprains, snake bites, unexplained swelling and pain, external injuries with swelling and stasis	Ma and Ye (2013), Medica et al. (2009), Xu and Huang (2017), Zhang and Du (2021)
Jino	Pediatric pneumonia, cholecystitis, tonsillitis, nephritis, gastritis, boils and carbuncles, pain and swelling, arthritis, and snake bites	Yang (2001)
De Ang	Epidemic encephalitis B, stomach pain, appendicitis, lymph node tuberculosis, tonsillitis, mumps, mastitis, snake and insect bites, sores and toxic swellings	Bureau (1990), Fang (2014)
Dai	Postpartum illnesses, menstrual irregularities, dysmenorrhea, amenorrhea, sore throat and swelling, mumps, swollen and painful submandibular lymph nodes, mastitis, carbuncles and boils, abdominal lumps and tumors, boils and abscesses	Cui and Tang (2007), Ma and Ye (2013), Medica et al. (2009), Zheng et al. (2019)
Zang	Lymph node tuberculosis	Zheng et al. (2019)
Hani	Mumps, bruises, and sprains, skin itching, toe ulceration (athlete’s foot), appendicitis, thyroid enlargement, pulmonary tuberculosis, bronchitis with cough, hemoptysis, hematemesis, epistaxis, bone tuberculosis, osteomyelitis, facial and lower limb edema, gout, etc.	Zheng et al. (2019)
Naxi	Sprains, nasopharyngeal carcinoma, gastritis, gastric ulcers, stomach pain and bloating, bites and stings, boils, carbuncles, furuncles, fractures, cervical lymph node tuberculosis, unexplained swellings and toxic conditions, mumps, leprosy	Zheng et al. (2019)
Pumi	Epidemic mumps, acute tonsillitis, hemoptysis, pulmonary tuberculosis, bronchiectasis, gastritis, chronic gastritis, gastrointestinal bleeding, vomiting blood, gastric and duodenal ulcers, chronic heart failure, aplastic anemia, snake bites, acute appendicitis	Zheng et al. (2019)
Jingpo	Gastric ulcers, bleeding from cuts	Zheng et al. (2019)
Achang	Clearing heat and detoxifying, reducing swelling, and relieving pain	Ma (2016); Medica et al. (2009)
Lahu	Epidemic mumps, high fever in children, tracheitis, leukemia, stomach pain, red and painful eyes, sore throat and swelling, fractures, snake bites	Zheng et al. (2019)
Wa	Rheumatoid arthritis, fractures, shoulder pain, joint pain, periarthritis of the shoulder, sprains and internal injuries, external injuries, silicosis, asthma, lung cancer, pulmonary tuberculosis, snake bites, insomnia, gastric ulcers, etc.	Zheng et al. (2019)
Buyi	Diarrhea	Luo and Sun (2013), Zheng et al. (2019)
Lisu	Snake and insect bites, sores and toxic swellings, mumps, mastitis, tonsillitis, etc.	Institute et al. (2021)
Shes	Various inflammations, snake bites, childhood convulsions, diphtheria and encephalitis B, carbuncles and boils, sore throat and swelling, pain from falls and impacts, anti-tumor, hemostasis, etc.	Mei (2018), Wang et al. (2018)
Tujia	Heart disease	Wang et al. (2018), Fang et al. (2007)
Zhuang	Snake bites, mastitis, unexplained swelling and toxic conditions, lymph node tuberculosis, mumps	Pan et al. (1993)
Dong	Treat sore throat and swelling, heal wounds and painful swelling, snake bites, convulsions and tremors, bruises and sprains, etc.	Li (2011)
Hui	Clearing heat and detoxifying, relieving cough and expectorating phlegm, reducing fever	Zhang (2008)

## 5 Medicinal plant preparations

Medicinal plant preparations have become an important part of natural medicine and are widely used worldwide. They have unique advantages in disease treatment and healthcare, and an increasing number of medical staff and patients recognize the value of medicinal plant preparations (Qiu et al., 2010). Members of the *Paris* spp. has been used as a botanical ingredient in some traditional medicinal formulations. It is combined with other botanical drugs to treat common pain and can also treat diseases such as psoriasis, eczema, and digestive system disorders. Medicinal plant preparations can be combined with different Chinese botanical drugs and proportions according to their efficacy to achieve the best therapeutic effect. The common names, scientific names, and Latin names of plants used in this article are listed in Table 3. The traditional *Paris* species medicinal plant preparation is listed in Table 4.

**TABLE 3:** The common names, scientific names, and Latin names of plants.

Common name	Scientific name	Latin name
Dudingzi	Jintiesuo	*Psammosilene tunicoides* W. C. Wu & C. Y. Wu
Pugongying	Dandelion	*Taraxacum mongolicum *Hand.-Mazz
Zihuadiding	Chinese violet	*Viola philippica* Cav
Songfengcao	Stinky Grass	*Boenninghausenia albiflora *(Hook.) Rchb. ex Meisn
Baizhi	Baizhi	*Angelica dahurica* (Fisch. ex Hoffm.) Benth. & Hook. f. ex Franch. & Sav
Zhizi	Gardenia jasminoides	*Gardenia jasminoides* J. Ellis
Weilingxian	Clematis	*Clematis chinensis *Osbeck
Mugua	Papaya	*Pseudocydonia sinensis* (Thouin) C. K. Schneid
Sanqi	Pseudo-ginseng	*Panax notoginseng *(Burkill) F. H. Chen ex C. H. Chow
Chonglou	Paris polyphylla	*Paris polyphylla* Sm
Gouteng	Uncaria	*Uncaria rhynchophylla* (Miq.) Miq. ex Havil
Tiannanxing	Tiannanxing	*Arisaema heterophyllum* Blume
Longdancao	Gentiana	*Gentiana cruciata* L
Taizishen	Pseudostellaria	*Pseudostellaria heterophylla* (Miq.) Pax
Xiakucao	Prunella vulgaris	*Prunella vulgaris* L
Zexie	Rhizoma alismatis	*Alisma plantago-aquatica* L
Maomei	Rubus parvifolius	*Rubus parvifolius* L
Mubiezi	Momordica cochinchinensis	*Momordica cochinchinensi*s (Lour.) Spreng
Dahuang	Huangbai	*Rheum palmatum* L
Mayaxiao	Mirabilite	*Natrii Sulfas*
Banxia	Pinellia ternata	*Pinellia ternata* (Thunb.) Ten. ex Breitenb
Bawangqi	Bawang Seven	*Impatiens textorii* Miq
Beishesheng	Back Snake Life	*Aristolochia tuberosa* C. F. Liang & S. M. Hwang
Wuya	Large-billed Crow	*Corvus macrorhynchus* Wagler
Heimao	Black cat	*Felis nigripes*
Tufuling	Tuckahoe	*Smilax glabra* Roxb
Daqingye	Isatis indigotica	*Isatis tinctoria* L
Baixian	Dictamnus dasycarpus	*Dictamnus dasycarpus* Turcz
Rendongteng	Honeysuckle vine	*Lonicera japonica* Thunb
Shandougen	Radix Sophorae Subprostratae	*Euchresta japonica *Benth. ex Oliv
Dihuang	Rehmannia glutinosa	*Rehmannia glutinosa* (Gaertn.) Libosch. ex Fisch. & C. A. Mey
Zicao	Lithospermum	*Lithospermum erythrorhizon* Siebold & Zucc
Huai	Sophora japonica	*Styphnolobium japonicum* (L.) Schott
Kushen	Sophora flavescens	*Sophora flavescens* Aiton
Difu	Summer cypress	*Bassia scoparia* (L.) A. J. Scott
Shechuang	Cnidium monnieri	*Cnidium monnieri* (L.) Spreng
Baifan	alum	KAl(SO4)_2_·12H_2_O
Huajiao	Zanthoxylum bungeanum	*Zanthoxylum bungeanum* Maxim
Huangbai	Chuanhuangbo	*Phellodendron chinense* C. K. Schneid
Yuxingcao	Houttuynia cordata	*Houttuynia cordata* Thunb
Xuanhuaqie	Solanum spirale	*Solanum spirale* Roxb
Tiancai	Beet	*Beta vulgaris *L
Tianhuafen	Trichosanthes rosthornii	*Trichosanthes rosthornii* Harms
Tianxianzi	Henbane	*Hyoscyamus niger* L
Gansui	Euphorbia kansui	*Euphorbia kansui Liou* ex S. B. Ho
Bohe	Mint	*Mentha canadensis* L

**TABLE 4:** Traditional genus *Paris* Medicinal Plant Preparations.

Traditional uses	Method of preparation	References
Ulcers and abscesses that have ruptured for a long time without closing the mouth	20 g Chonglou, 10 g Dudingzi, powder together, add 50 g Vaseline, mix well and apply externally	Jiang and Xiao (2021)
Sore pain, unnamed swelling toxin mastitis and mumps	Take 10–15 g of dry powder from Chonglou, mix with sweet rice wine, steam or boil in water, and drip the wine as a guide; Alternatively, 10 g of Pugongying, Zihuadiding, and Songfengcao can be added and boiled in water before consumption. Alternatively, mix 20 g of Baizhi and 10 g of Zhizi with powder and apply as a paste	Jiang and Xiao (2021)
Brain tumor	30 g of Chonglou, 30 g of Weilingxian, 9 g of Mugua, decoct in water and swallow 3 g of Sanqi powder, 1 dose per day	Chang (1996)
Nasopharyngeal carcinoma	Chonglou 50–100g, Gouteng 15g, Shengnanxing 50–150g, Longdancao, Taizishen, Xiakucao 15 g each, Zexie 50g, Maomei 100 g. 1 dose per day	Chang (1996)
Esophageal cancer	12 g of Chonglou, 9 g each of fried Dahuang and Mubiezi, 12 g of Mayaxiao, 0.3 g of Banxia, a total of fine powder, refined into 3 g of pills, gradually containing 3–4 pills per day	Chang (1996)
Reduce swelling and neutralize snake and insect venom	Paired with Banxia, Nanxing, and Bawangqi, apply a combination of flushing and velvet externally	Institute Of Traditional Chinese Medicine and Chinese Academy Of Sciences (1960)
Treat those with positive results in Jingfeng	Mix with Gansui, stir fry Huafen until charred and ground, and add Bohe soup. Take 5 min each time	Institute Of Traditional Chinese Medicine and Chinese Academy Of Sciences (1960)
Acute gastroenteritis	Take an appropriate amount of Chonglou and Zhushalian, decoct in water and take orally	Zhao and Yang (2017)
Haemorrhoids	Take appropriate amounts of Chonglou, Black Cat Head (without fur), and Crow Head (without fur), crush them, and apply them to the affected area	Zhao and Yang (2017)
Psoriasis vulgaris	Tufuling soup is made by combining the ingredients of Daqingye, Rendongteng, Baixianpi, and Shandougen to clear heat and detoxify; Sheng Dihuang, Zicao, and Huai flowers have good effects in clearing heat and cooling blood, and treating blood heat psoriasis	Chen and Jiang (2017)
Eczema	15 g of Chonglou mixed with 10 g of Kushen, Difuzi, Shechuangzi, and Huangbai, 9 g of alum, and 6 g of Huajiao decoction	Chen and Jiang (2017)
Wind toxin induced swelling	30 g each of Chonglou, Mubeizi (shell removed), and Banxia, finely ground into powder and mixed with vinegar for application	Ma and Ye (2013)
Scabies swelling	30 g of fresh Chonglou and 30 g of Yuxingcao, mashed and applied externally to the affected area, once a day	Ma and Ye (2013)
Mouth and tongue sores, sore throat	Take equal amounts of Chonglou, Xuanhuaqie roots, and Tiancai root, dry and grind them finely, mix well, and serve with warm water, 3–5 g each time	Ma and Ye (2013)
Ulcers, abscesses, toxins, scrofula, and boils	Take 9 g of Chonglou and 30 g of Pugongying, decoct in water and take orally; Apply 30 g of Chonglou and Tianhua powder for external use, and 15 g of Tianxianzi powder. Mix the powder with boiling water and apply it to the affected area	Ma and Ye (2013)

## 6 Chemical constituents

*Paris* spp. have various metabolites, among which steroidal saponins are the most active. The rhizome, as the central medicinal part, has attracted the attention of many plant chemistry researchers. At the same time, some chemical constituents have also been found in other parts of *Paris* species, enriching the plant chemical research of the medicinal materials of *Paris* spp., which helps to clarify the practical metabolites of *Paris* spp. and expand global pharmaceutical resources. A total of 431 compounds have been isolated from *Paris* species. In this article, we will introduce the names, medicinal parts, and effects of each type of metabolite in Supplementary Table 5. Compiling a list and categorizing compounds can facilitate researchers in locating macrocyclic compounds of the same type, enhance research efficiency, optimize database management, and ensure data integrity and accuracy. And the different types of chemical structures in Supplementary Tables 6–17.

### 6.1 Steroidal saponins

Steroidal saponins are formed by the condensation of steroidal sapogenins with sugars. The steroidal sapogenin consists of 27 carbon atoms forming six rings labeled A, B, C, D, E, and F, and the molecule contains multiple hydroxyl groups, most of which have a β-configured hydroxyl group at the C-3 position. There are three chiral carbon atoms in the E and F rings, which are C-20, C-22, and C-25. The absolute configurations of C-20 and C-22 are S and R, respectively, while C-25 can have either the S or R configuration (Tian, 2022). Based on the configuration of C-25 and the cyclization state of the F ring, Steroidal saponins are divided into four types: spirostanol type, isospirostanol type, furostanol type and Modified spirostanol type. Saponin aglycones are usually glycosylated at the C-3 position and the C-1 position to form glycosides, and they can also be glycosylated at the C-7 position and the C-26 position. The common sugar moieties that compose saponins are Glu, Rha, Xyl,Fuc and Ara, and Api and Gal.

**FIGURE 3:**
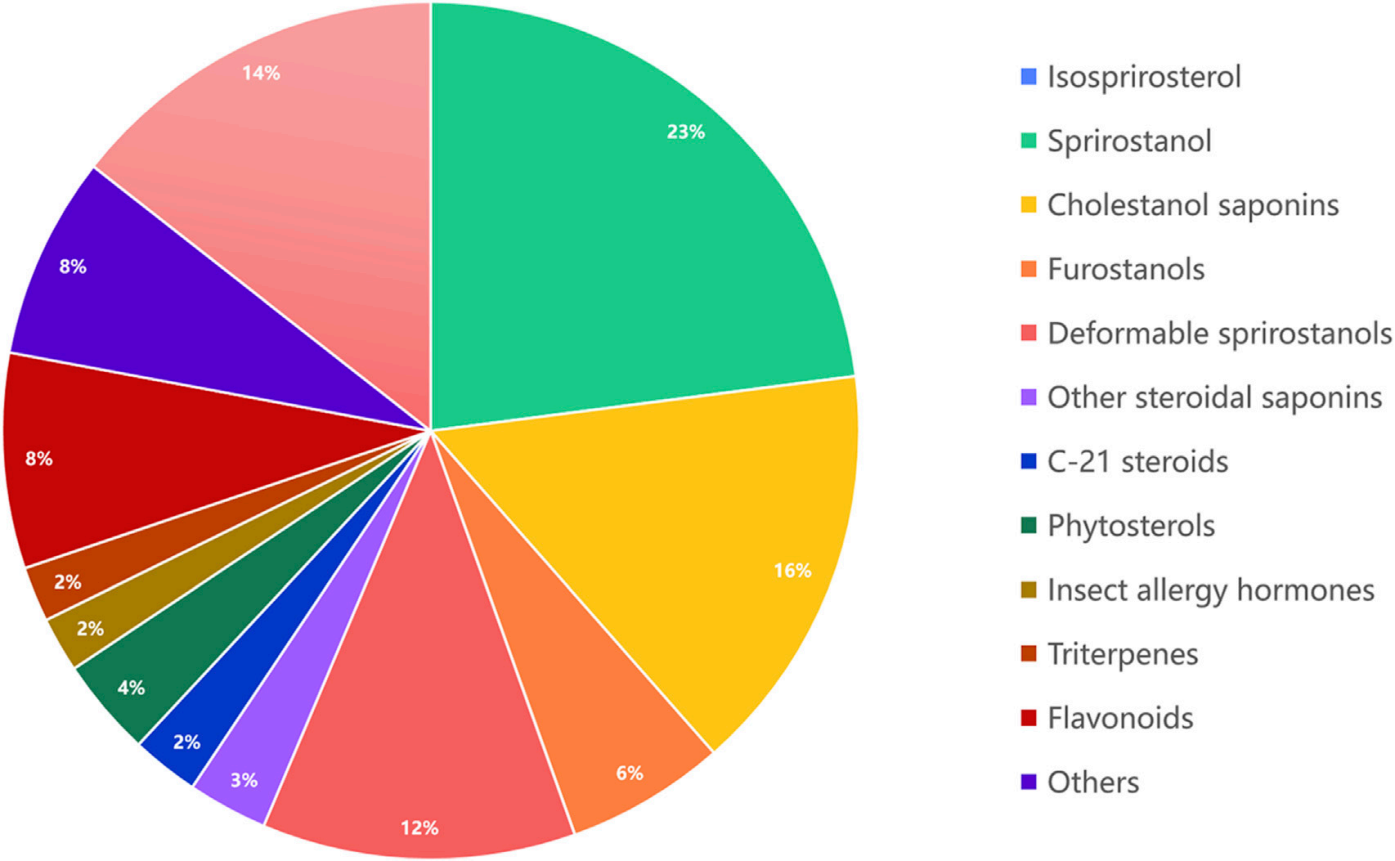
The composition and proportion of chemical components in the *Paris* spp.

It has been proven that steroidal saponins are the main metabolites of *Paris* spp. and possess a wide range of pharmacological properties, including anti-tumor, anti-inflammatory, anti-angiogenic, anti-metastatic, and hemostatic characteristics. In addition, research has demonstrated that steroidal saponins exhibit anti-cancer activity against various cancers through different molecular mechanisms, including apoptosis, cell cycle arrest, and inhibition of migration and invasion (Liu et al., 2023). Combining the above list of compounds, Figure 3 displays the metabolite and proportion of chemical components in the *Paris* spp.

#### 6.1.1 Isosprirostanol saponins

The C-25 position of the isospirostanol-type steroidal saponins has a methyl group that is positioned on the horizontal bond of the F ring plane, which is *α*-oriented and has an absolute configuration of R. The isospirostanol type steroidal saponins are one of the main anti-tumor active components of *Paris* spp., with the representative metabolite being polyphyllin Ⅰ (Tian, 2022)、polyphyllin Ⅱ(Hua, 2015)、diosgenin (Jing et al., 2017)、polyphyllin Ⅵ (Tian, 2022)、polyphyllin Ⅶ (Hu, 2022)、polyphyllin H (Tian, 2022)、polyphyllin E (Man et al., 2011)、polyphyllin A (Qin et al., 2012) and so on.

The variety of isospirostanol-type saponins detected in *Paris* species is the most abundant, with a total of 431 metabolites identified, of which 99 are spirostane-type saponins. Wang et al. (2020) used ultra-high-performance liquid chromatography - quadrupole time-of-flight mass spectrometry (UHPLC-QTOF-MS) for the identification of *P. polyphylla*, and a total of 222 compounds were identified, including 77 isospirostanol type saponins.

Duyen et al. (2022) found that paris saponin II can serve as an anti-cancer drug. Paris saponin II significantly increases the levels of P53 and Bax proteins, inducing apoptosis in MCF-7 cells and regulating cell cycle arrest.

#### 6.1.2 Sprirostanol saponins

Spirostanol saponins are a class of oligosaccharides derived from spirostanol compounds, with the main aglycones of spirostanol saponins being 27-carbon cyclopentane-hydrogenated phenanthrene steroid compounds. Spirostanol-type steroidal saponins are the main active metabolites of the famous medicinal material of *Paris* spp., and they exhibit significant anti-cancer (Qin et al., 2020), anti-tumor (Hu et al., 2023), and antibacterial (Duan et al., 2023) activities. These saponins are also listed as quality control ingredients in many countries’ *Paris* species.

Liu (Liu, 2018) found in their research that the compound N-20 has a certain inhibitory effect on glioma cells, demonstrating the anti-tumor effect of spirostane saponins. Qin et al. (Qin et al., 2012) experimentally discovered that the spirostanol saponins disoseptemloside H was the first to be isolated from *Paris* spp. Chonglouoside SL-6, which contains a trisaccharide part at the C-1 position, has a MIC value of 3.9 L g/ml and exhibits good activity against *propionibacterium acnes*.

#### 6.1.3 Cholestane saponins

Cholestane saponins are a steroidal saponins class formed by combining sterane compounds with sugars. The aglycone of cholestane saponins is a derivative of spirostane, and the cholestane saponin aglycone is structurally characterized by an incompletely opened F-ring structure of the spirostane saponin, typically composed of 27 carbon atoms. Two new cholestane saponin compounds, parispolyoside A and parispolyoside E, were isolated from *P. polyphylla* var. *Chinensis* (Guan et al., 2024). Qin et al. (Qin et al., 2016) also found that the compound chonglouoside SL-19 is a new cholestane saponin, with only a tetrasaccharide chain at the C-3 position, which is the first to be isolated from members of the genus *Paris*. These newly discovered cholestane saponin compounds have increased the structural diversity of steroidal saponins in *Paris* spp*.*

#### 6.1.4 Furosterol saponins

Furosterol saponins are widely distributed in higher plants such as the Asparagaceae, Alliaceae, Liliaceae, Dioscoreaceae, and Solanaceae families. As common monosaccharide chain spirostane saponin precursors, furosterol saponins are mainly found in the leaves and metabolically active organs of plants, and they also exist in other organs of plants (Zheng, 2005). The most distinctive structural feature of the furosterol saponin aglycone is the fully opened F-ring structure of the spirostane saponin.

Furosterol saponins are also present in the rhizomes of *Paris* species and possess anti-tumor and anti-cancer activities. Liu et al. (Liu et al., 2016) isolated four new furosterol saponin compounds, padelaosides C–F, from the rhizomes of *P. delavayi*. Not only that, but they also found that padelaosides D and padelaosides F exhibit certain cytotoxicity to human glioma cells, with MIC values ranging from 15.28 to 16.98 μmol/L. In addition, Guan et al. (Guan et al., 2024) have found that furosterol saponin compounds such as parpetioside C, Th, parisyunnanoside A, and pseudoprotogracillin show moderate cytotoxic activity against HepG2 cells, with IC_50_ values in the range of 9.43–24.54 μM.

#### 6.1.5 Deformable spirostanol saponins

The structural characteristics of deformable spirostanol saponins are mainly reflected in the deformation of their F-ring, where the F-ring of the aglycone of deformable spirostanol saponins is deformed into a furan ring. A notable feature of the deformable spirostanol saponins is the opening of the F-ring and the frequent hydroxyl or methyl substitution at the carbon 26 position. Zheng et al. (Zheng et al., 2023) isolated the deformable spirostanol saponin compound aculeatiside A from the aerial parts of *P. polyphylla* var. *chinensis*, which was the first time this compound was isolated from the genus of *Paris*. Qin et al. (Qin et al., 2016) obtained new deformable spirostanol saponin compounds, chonglouoside SL-10 and chonglouoside SL-13, from the stem and leaf parts of *Paris polyphylla* var. *yunnanensis*. Moreover, abutiloside L was found to have inhibitory effects on two human cancer cell lines (HepG2 and HEK293). The discovery of these metabolites indicates that the aerial parts of *Paris* species are rich in saponin compounds, adding to the structural diversity of steroidal saponins in *Paris* spp.

#### 6.1.6 Other steroidal saponins

Due to C-25 not being a chiral carbon atom and not being classified as spirostane or furostane saponins, they are identified as other steroidal saponin compounds. Jiang et al. (Jiang et al., 2022) have shown in their research that two new compounds, Parisyunnanoside K and Parisyunnanoside L, were isolated from *P. polyphylla* var. *yunnanensis*. Furthermore, in 2023, Liu et al. (Liu et al., 2023) discovered three new other steroidal saponin compounds, parisverticillatoside B-D. The discovery of these new metabolites has increased the structural diversity of steroidal saponins within *Paris* spp.

### 6.2 C-21 steroids

C-21 steroids are a group of steroidal derivatives containing 21 carbon atoms, primarily based on the basic skeleton of pregnane or its isomers, and research has found that these metabolites also have antimicrobial activity. Hu et al. (Hu et al., 2023) isolated a new C-21 steroid compound, paristenoids C, from the *P. polyphylla* var. *stenophylla*., expanding the diversity and complexity of *Paris* species saponin family. In 2013, Qin et al. (Qin et al., 2013) isolated two new compounds, chonglouoside SL-7 and chonglouoside SL-8, from the stem and leaves of *P. polyphylla* var. *yunnanensis*. In addition, the compound chonglouoside SL-7 also has antimicrobial activity against *Propionibacterium acnes*, with an MIC_50_ value of 31.3 μg/mL.

### 6.3 Phytosterols

Phytosterols are naturally occurring triterpenoid compounds found in plant cell membranes and serve as precursor substances for the biosynthesis of various hormones, vitamin D, and steroid compounds. They possess a range of benefits, including cholesterol reduction, blood lipid-lowering, treatment of cardiovascular diseases, and anti-inflammatory and anti-cancer effects (Wang et al., 2024). Phytosterols are triterpenoid compounds with cyclopentane polyhydrophenanthrene as the main skeleton (sterol nucleus), typically containing 28 to 29 carbon atoms, forming three six-membered rings and one five-membered ring. In the molecular structure, the hydroxyl group at the C-3 position is the main active group of phytosterols. Most phytosterols have a double bond at the C-5 position, and an R group containing 8 to 9 carbons is connected at the C-17 position. Structurally, phytosterols are similar to cholesterol, with the difference only in the R group connected at the C-17 position. Based on the different side chains, phytosterols can be divided into 4-desmethylsterols, 4-methylsterols, and 4,4′-dimethylsterols, with the most studied being 4-desmethylsterols, including β-sitosterol, campesterol, brassicasterol, and stigmasterol, among others (Lou et al., 2018).

### 6.4 Triterpenes

Triterpenes, a class of secondary metabolites extracted from plants, possess unique chemical structures composed of triterpenoid or steroidal aglycones combined with one or more sugar molecules. They play a significant role in the pharmaceutical, fragrance, and cosmetic industries and are also considered a key part of plant defense mechanisms due to their resistance to bacteria, fungi, and insects (Pan et al., 2023). A total of 35 triterpene compounds have been isolated from *Paris* species*,* including lupeol (Liu et al., 2014), which is a lupane-type triterpene saponin, cussonoside B (Zhang et al., 2014) which is a cucurbitacin, and (23Z)-9,19-cycloart-23-ene-3α,25-diol, which is a tetracyclic triterpene saponin.

### 6.5 Flavonoids

Flavonoids are also present in *Paris* spp. Two benzene rings and a central three-carbon structure typically form these compounds. Based on the attachment sites of the heterocyclic ring, the degree of oxidation, and the unsaturation of the three-carbon chain, they are divided into the following subcategories: flavones, flavonols, flavanones, flavanols, isoflavones, anthocyanins, and chalcones (Li, 2023). Flavonoids are important secondary metabolites within plants and play a key role when plants face biotic and abiotic stresses. By participating in the plant’s resistance mechanisms, flavonoids help to enhance the plant’s stress resistance, thereby ensuring the healthy growth of the plant (Zhu et al., 2024).

### 6.6 Other compounds

In addition to the aforementioned chemical components, there are 51 chemical constituents involving fatty acids palmitic (Xu et al., 2023), CH_3_(CH)_14_COOH (Cui, 2006)), gallic acid (Hua, 2015), vanillin (Qin et al., 2016), and other compounds. In 2007, Wang et al. (Wang et al., 2007) isolated two new compounds, parispolyside F and parispolyside G, from the rhizomes of *P. polyphylla* var. *yunnanensis*. These non-steroidal saponin compounds greatly enrich the diversity of the chemical constituents of *Paris* species.

### 6.7 Insect allergenic hormones

*Paris* spp. mainly contain metabolites such as *β*-ecdysone (Hu, 2022), calonysterone (Guo et al., 2021), *β*-ecdysterone (Yang et al., 2024), 5-hydroxyabutastorone (Tang et al., 2018). Among these, *β*-ecdysone is distributed in various members of the genus *Paris*, including *P. polyphylla* var. *yunnanensis*, *P. polyphylla* var. *japonica*, and *P. fargesii*.

## 7 Pharmacological effects

**FIGURE 4:**
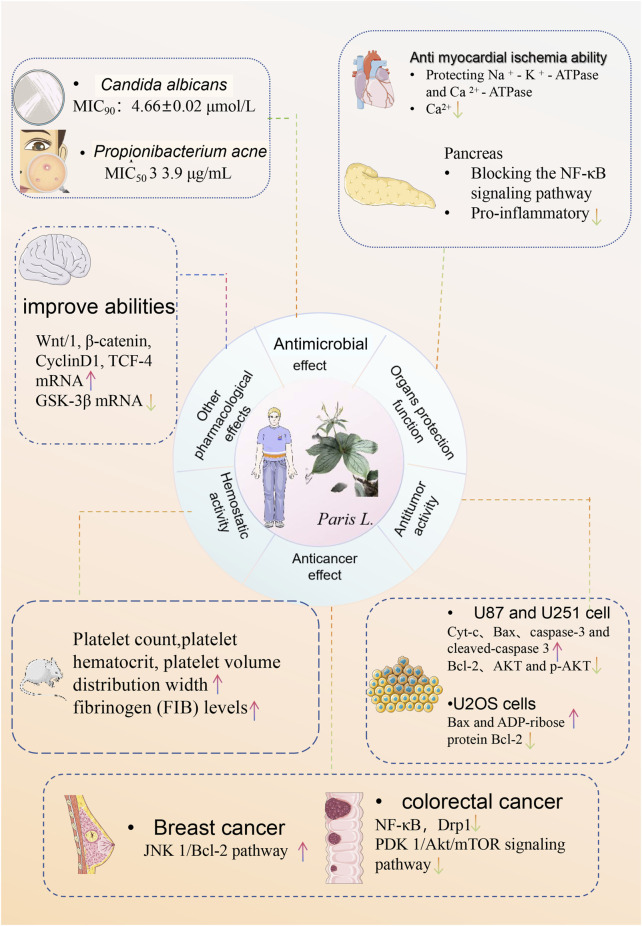
The main pharmacological effects of *Paris* spp.

**FIGURE 5:**
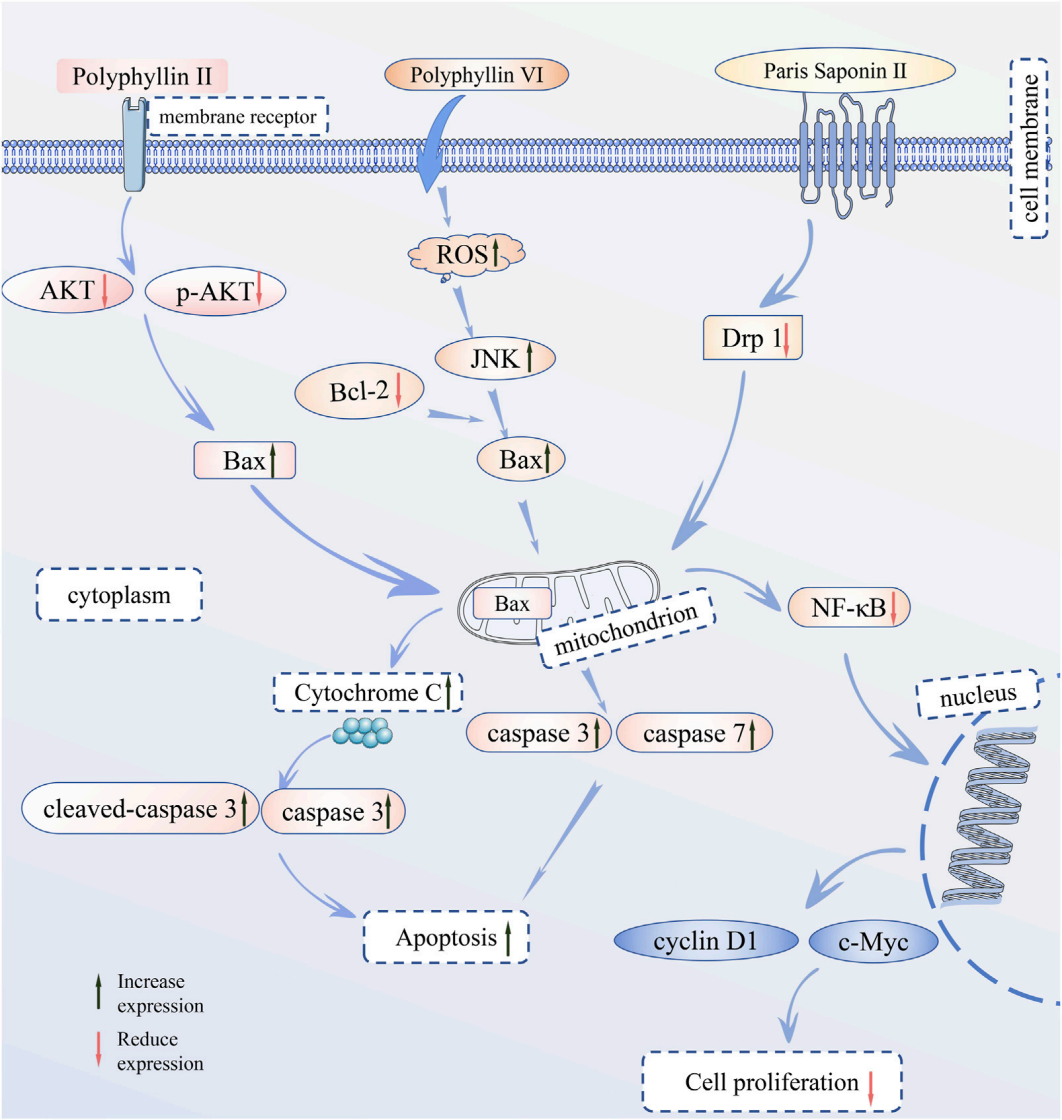
The Main mechanisms of *Paris* spp.

Scientific research has illuminated that steroidal saponins, as the main active metabolites in *Paris* species, exhibit a variety of pharmacological effects, such as anti-tumor (Li et al., 2021), anti-angiogenesis (Zhang et al., 2021a), immune response modulation (Liu et al., 2015), and hemostasis. *Paris* species is used to produce medicines such as “Yunnan Baiyao” and “Gongxue Ning,” demonstrating significant therapeutic effects in hemostasis, anti-inflammation, and the treatment of injuries from falls and impacts. Further research has found that steroidal saponins can combat cancer through multiple molecular mechanisms, including inducing apoptosis in cancer cells, blocking the cell cycle (Duyen et al., 2022), and inhibiting the migration and invasion of cancer cells (He et al., 2014). Based on these findings, steroidal saponins from members of the genus *Paris* are expected to become candidate substances for developing anti-cancer drugs (Liu et al., 2023). However, the specific mechanisms of some pharmacological effects of *Paris* spp. are not yet fully understood, and more clinical trials and systematic evaluations are needed for the translation from laboratory research to clinical application.The main pharmacological effects of *Paris* species are shown in Figure 4. The Main mechanisms of *P. polyphylla* in the treatment of various cancers are shown in Figure 5.

### 7.1 Anti-tumor effects

Research indicates that *P*. *polyphylla* Smith extracts can inhibit the proliferation of various tumor cells. Steroidal saponins can directly act on tumor cells to exert anti-tumor effects and can also intervene in tumor growth and migration by mediating apoptosis or inhibiting proliferation mechanisms (Zhang et al., 2021b). Besides, studies from both domestic and international sources have shown that the anti-tumor mechanism of steroidal saponins mainly works by inducing apoptosis and autophagy, inhibiting cell growth and proliferation, blocking the cell cycle, and inhibiting tumor metastasis (Du et al., 2024). These findings provide an important foundation for further research and the development of new anti-cancer therapies.

Western blot results show that Polyphyllin Ⅱ inhibits the growth of U87 and U251 cells, and its mechanism of action is that Polyphyllin Ⅱ promotes the expression of cytochrome c, Bax, caspase-3, and cleaved-caspase 3, while reducing the expression of Bcl-2, AKT, and p-AKT (Cheng et al., 2021). In 2019, Yuan et al. (Yuan et al., 2019) found that Polyphyllin Ⅵ induces apoptosis in human U2OS cells by upregulating the pro-apoptotic protein Bax and (ADP-ribose) polymerase and downregulating the anti-apoptotic protein Bcl-2. Integrating the aforementioned research, steroidal saponins can inhibit the growth of tumor cells by mechanisms such as upregulating Bax and downregulating Bcl-2.

*In vitro* tumor experiments have significant advantages in clarifying the mechanisms of drugs, ensuring reproducibility and controllability, and conducting preliminary screening and mechanism studies. They can efficiently verify the anti-tumor effects and provide a theoretical basis for subsequent research. However, their shortcomings lie in the inability to fully simulate the complex *in vivo* environment, the lack of consideration for drug delivery and bioavailability, and limited assessment of toxicity and safety. Therefore, further validation of their efficacy and safety is needed through *in vivo* experiments and clinical trials.

### 7.2 Anti-cancer effects

Rhizoma paridis total saponins are the main metabolites of *Paris* species, and studies have revealed that this substance has significant anti-tumor effects on various solid tumors such as breast cancer, colorectal cancer, and glioma. Its mechanism of action may involve multiple aspects, including directly exerting toxicity on tumor cells, modulating the body’s immune response, inhibiting the formation of tumor blood vessels, and reducing the resistance of tumor cells to therapeutic drugs (Zhong et al., 2019).

In 2022, Liu et al. (Liu et al., 2022) found that Polyphyllin D could induce apoptosis in breast cancer cells by activating the JNK 1/Bcl-2 pathway. *P. polyphylla* Smith extract inhibits the growth and proliferation of human esophageal cancer ECA 109 cells by increasing the expression of Cx26 mRNA and protein and decreasing the expression of Bcl-2 (Li et al., 2012). Mitochondria play an important role in the process of apoptosis; Li et al. (Chen et al., 2019) demonstrated that the mechanism involves the connection between NF-*κ*B and mitochondrial fission. Paris Saponin Ⅱ inhibits the activation of NF-*κ*B by suppressing Drp1, thereby downregulating the expression of cyclin D1 and c-Myc genes, thus achieving the therapeutic effect on colorectal cancer. In addition, Polyphyllin I inhibited the PDK1/Akt/mTOR signaling pathway in HGC-27 cells, inducing apoptosis (He et al., 2019). These cancer cell experiments were conducted *in vitro* but cannot fully simulate *in vivo* conditions. Therefore, their results cannot directly predict *in vivo* therapeutic effects. Further *in vivo* studies, such as animal models or clinical trials, are needed to validate the therapeutic efficacy and safety and to assess their impact on cancer progression and patient outcomes.

*In vivo* and *in vitro*, Polyphyllin Ⅶ inhibits angiogenesis and metastasis of HepG2 cells by downregulating the NF-*κ*B/MMP-9/VEGF pathway (Zhang et al., 2021a). This finding indicate that Polyphyllin Ⅶ has the potential to serve as a promising therapeutic agent for hepatocellular carcinoma (HCC).Hong et al. found that polyphyllin Ⅰ inhibits tumor growth and induces apoptosis of nasopharyngeal carcinoma cells both *in vitro* and *in vivo* by downregulating lncRNA-ROR, which subsequently upregulates the P53 signaling pathway (Hong et al., 2019). In addition, Niu et al. (Niu et al., 2020) found that Polyphyllin Ⅱ increases the expression level of E-cadherin and decreases the expression levels of N-cadherin, snail family transcriptional repressor 2, twist family bHLH transcription factor 1, matrix metalloproteinase MMP 2, and MMP 9, significantly inhibiting the migration and invasion of human bladder cancer cells. Formosanin C inhibits lung cancer growth through a novel mechanism involving the downregulation of MCT 4 and CD 147 expression, blocking lactate export and mitochondrial function disruption (Li et al., 2023).

The aforementioned studies demonstrate that *Paris* species exhibits significant therapeutic efficacy against a variety of cancers, making it a highly promising candidate for the development of novel anticancer strategies. However, it is important to note that while members of the genus *Paris* may protect one organ, it is uncertain whether it might have adverse effects on other organs, as research in this area is currently lacking. Further studies are needed to elucidate the pharmacological actions and complex mechanisms of sophoridine under these pathological conditions.

### 7.3 Anti-inflammatory and analgesic effects

*P. polyphylla* possesses the effects of clearing heat and detoxifying, reducing swelling and relieving pain, cooling the liver, and calming convulsions, and is one of the important ingredients in many traditional Chinese patent medicines (such as Yunnan Baiyao, Gongxue Ning, and Ji De Sheng Snake Medicine Tablets). It is commonly used for symptoms like carbuncles and swelling, sore throat and pain, snake and insect bites, and fall injuries (Ding et al., 2018). Literature reports that the anti-inflammatory effect of *P. polyphylla* Smith extracts is significant and has good clinical effects, often used to alleviate oral pain and swelling caused by gum, periodontal, and mucosal inflammation (Xiao et al., 2021). He et al. (He et al., 2019) found that rat models were established using ALCT surgery and divided into groups: OA model group, Polyphyllin VII groups (25, 50, 100 mg/kg), Diacerein Capsule group (50 mg/kg), and sham surgery group (normal saline).*P. polyphylla* can also treat osteoarthritis. Polyphyllin Ⅶ achieves the treatment of osteoarthritis by reducing TNF-*α*, IL-6, and IL-1*β* levels in knee joint fluid and inhibiting IL-1*β*, MMP9, and ADAMTS-5. Besides, increasing the dose of Polyphyllin VII enhances its efficacy. Specifically, rats treated with 100 mg/kg of Polyphyllin VII exhibited greater movement distance compared to those treated with 50 mg/kg of diacerhein capsule.

Research has found that IL-1*β* is a key factor in inducing osteoarthritis, capable of exacerbating cellular inflammatory damage, inducing apoptosis, and ultimately leading to the destruction of articular cartilage (Shi et al., 2020). Li et al. (Li et al., 2021) found that compared with the control group, the IL-1*β* group showed increased protein expression of *β*-catenin, c-myc, and iNOS in articular chondrocytes, decreased cell viability, and elevated apoptosis rate and expression of cleaved Caspase-3. Additionally, the secretion levels of IL-6, TNF-*α*, IL-8, and NO were higher in these cells. Compared with the IL-1*β* group, the low-, medium-, and high-dose groups of Polyphyllin Ⅷ exhibited gradual improvements in these indicators, with higher doses correlating with higher cell viability. The above results indicate that Polyphyllin Ⅷ reduces apoptosis and secretion of inflammatory mediators induced by Ⅱ-*β* in chondrocytes by inhibiting the Wnt/*β*-catenin signaling pathway. Polyphyllin Ⅶ reduces the production of NO and PGE2 in LPS-induced RAW264.7 cells by inhibiting the NF-*κ*B and MAPKs pathways while also reducing the protein and mRNA expression of pro-inflammatory cytokines (TNF-*α*, IL-1*β*, and IL-6) and enzymes iNOS, COX-2, and matrix MMP-9. Additionally, Polyphyllin Ⅶ significantly inhibits dimethylbenzene-induced ear edema in mice and cotton ball-induced granuloma formation, as well as exhibiting inhibitory effects on inflammation and toxicity in zebrafish embryos induced by LPS and CuSO4 (Zhang et al., 2019).

The experimental method involves establishing a mouse model of bronchial asthma through sensitization with ovalbumin, followed by treatment with total saponins of *Paris polyphylla* at doses of 2.5 mg/kg and 10 mg/kg. Tan et al. found that oral administration of total saponins from *P. polyphylla* can treat allergic asthma;.its mechanism of action involves suppressing the production of the cytokine IL-4 by Th2 cells and restoring the reduced cytokine IFN-*γ* by Th1, thereby restoring the local Th1/Th2 cytokine balance, indicating that total saponins of *P. polyphylla* can reduce airway inflammation and cellular infiltration and decrease the total IgE level, effectively alleviating the symptoms of allergic asthma (Tan et al., 2017).

In the experiments mentioned above, *in vitro* experiments are conducted for the preliminary screening of drugs. Subsequent *in vivo* experiments simulate physiological environments to validate efficacy, toxicity, and metabolic properties, yielding results that are more aligned with real-world conditions. The combination of both approaches allows for mutual verification, clarifying dose-response relationships, therapeutic effects, and toxicity, thereby providing a foundation for preclinical research.

### 7.4 Antimicrobial effect

The steroidal saponins from *P. polyphylla* exhibit antifungal activity. Research has found that both the steroidal saponins and extracts of *P. polyphylla* Smith have an inhibitory effect on various strains of *Candida* albicans, including those resistant to fluconazole. Ophiopogonin C′ has shown good antibacterial activity, with an MIC_90_ of 4.68 ± 0.01 μmol/L for *Candida* albicans and 4.66 ± 0.02 μmol/L for fluconazole-resistant strains of *Candida* albicans (Duan et al., 2023). Qin et al. (Qin et al., 2013) found that the compounds Chonglouoside SL-7 and dumoside, isolated from members of the genus *Paris*, have certain antibacterial activity against Propionibacterium acnes, with MIC values of 31.3 and 3.9 μg/mL, respectively.

In addition, compounds 2, 3, and 4 have been proven to have certain antibacterial activity, with their minimum inhibitory concentrations against *Saccharomyces cerevisiae* being 2.5 mg/mL, 0.6 mg/mL, and 0.6 mg/mL, respectively (Zhu et al., 2011). Qin et al. (Qin et al., 2018a) research indicates that *P. polyphylla* var. *yunnanensis* can serve as a supplementary medication for globally susceptible fungal diseases. The *P. polyphylla* Smith extracts exhibit certain antifungal activities, such as TSSAPs and TSSRs demonstrated potent antifungal activity against *Candida* albicans (5314) and *Candida* albicans (Y0109), exhibiting MIC values of 5.15 μg/mL and 10.3 μg/mL, respectively. Additionally, the study also discovered that the four spirostanol saponins, including Paris saponin I, Paris saponin Ⅴ, Dioscin, and Paris saponin Ⅱ, exhibited pronounced antifungal effects against *Candida* albicans (5314) and *Candida* albicans (Y0109). They achieved an MIC value of 1.95 μg/mL, outperforming the antifungal susceptibility of the standard drug voriconazole.

Given that *in vitro* antimicrobial experiments cannot replicate the host environment or assess the evolution of drug resistance, they may lead to biased antimicrobial efficacy and lack evaluations of compound metabolic stability and *in vivo* distribution. Therefore, it is necessary to combine *in vivo* experiments and clinical studies to comprehensively validate the therapeutic potential and safety.

### 7.5 Organ protective effects

Evidence from clinical studies and animal experiments suggests that *Paris* species exhibits multifaceted benefits in anti-inflammation, including combating pathogenic microorganisms, regulating the immune system, providing antioxidant protection, and safeguarding organ function (Xiong et al., 2016). Steroidal saponins and their extracts from *Paris* species play a crucial role in organ protection. In this study, a SAP (severe acute pancreatitis) rat model was created via retrograde injection of 5% sodium taurocholate. Dexamethasone (2 mg/kg) served as the positive control, and Polyphyllin Ⅶ was tested at low (50 mg/kg) and high (150 mg/kg) doses. Results indicated that high-dose Polyphyllin Ⅶ had similar efficacy to dexamethasone, both outperforming the low-dose group. Wan et al. Found that Polyphyllin Ⅶ reduces the release of pro-inflammatory factors by blocking the NF-*κ*B signaling pathway, effectively protecting against lung injury caused by severe acute pancreatitis (SAP) (Wan et al., 2022). Li et al. isolated neonatal rat cardiomyocytes and subjected them to an anoxia-reoxygenation injury, which mimics *in vivo* ischemia-reperfusion injury, in the presence or absence of *Paris polyphylla* Smith extract (EPPS) or diltiazem (positive control).The direct protective effect of the extract from *P. polyphylla* Smith on cardiomyocytes is anti-ischemic to the heart, and the higher the concentration of the extract, the better the effect. Its mechanism being the protection of Na^+^-K^+^-ATPase and Ca^2+^-ATPase activities while inhibiting excessive intracellular calcium accumulation. This mechanism helps protect isolated neonatal rat cardiomyocytes from damage during hypoxia and reoxygenation (Li et al., 2011).

*Paris* species has a significant protective effect on the kidneys of rats with membranous nephropathy, and its therapeutic effect is comparable to tri-pterygium glycosides. In the experiment, a rat MN model was successfully established using cationized bovine serum albumin (C-BSA), and the rats were randomly assigned to the model group, the *Paris* spp. treatment group, and tripterygium glycosides treatment group (as a positive control). *Paris polyphylla* extract was decocted to 0.25 g/mL. Rats in the *Paris polyphylla* group were gavaged at 2 g/(kg·d), and tripterygium glycosides were gavaged at 10 mg/(kg·d). The experimental results showed that *Paris* species treatment group and the tripterygium glycosides treatment group could effectively alleviate proteinuria and hypercholesterolemia caused by the model. In addition, both treatments significantly reduced the fluorescence intensity of IgG and C3 in the glomeruli and decreased the expression level of fibronectin (FN) mRNA. These changes indicate that members of the genus *Paris* and tripterygium glycosides can improve the overall condition of rats and have a mitigating effect on renal pathological damage (Huang and Liu, 2007).

In the aforementioned experiments, the drug concentrations exceeded those of the positive controls, potentially increasing cytotoxicity or inducing non-specific reactions that could mask the true mechanisms of action. Many extracts are complex mixtures, and interactions among active components can affect efficacy and safety. Therefore, these factors should be considered in experimental design and result interpretation to ensure the accuracy and reliability of the outcomes.

### 7.6 Hemostatic activity

*Paris* spp. is an ingredient in medicines such as “Yunnan Baiyao” and “Jidesheng Snake Waist Tablets”, significantly affecting wound healing. It enters the liver meridian, which can reduce swelling, relieve pain, resolve stasis, and stop bleeding. The hemostatic mechanism promotes the conversion of fibrinogen to fibrin, activating coagulation factors through enzymes to generate thrombin, forming a clot, and achieving hemostasis (Ding et al., 2021). The hemostasis process requires the direct involvement of platelets, coagulation factors, and blood vessels. Luo et al. (Luo et al., 1988) found that steroidal saponin C can significantly shorten the coagulation time, and its hemostatic mechanism may be to promote the function of the endogenous coagulation system and induce vasoconstriction.

Furthermore, the extract of *Paris polyphylla* Smith rhizome has hemostatic effects on mice. Studies have shown that both the aqueous and alcoholic extracts can significantly reduce the hemostasis time and decrease the amount of bleeding, and the hemostatic effect is directly proportional to the dose. In particular, the alcoholic extract has a more pronounced hemostatic effect. When the alcoholic extract is administered intragastrically at a dose of 15 g/kg and observed 2 h later, the hemostatic effect is optimal. The hemostatic mechanism may involve increasing the number of platelets, platelet crit, platelet volume distribution width, and the fibrinogen (FIB) level, thereby promoting the hemostasis process in mice (Peng et al., 2019).

The experiments comprehensively evaluated the hemostatic effects of *Paris polyphylla* extract, clarified its dose-dependence, and revealed the underlying hemostatic mechanisms. However, the complex composition of the extract and the higher drug concentrations compared to positive controls may compromise the accuracy of the results. Additionally, the studies lacked consideration of long-term effects and individual differences. Future research should focus on isolating and purifying active components, optimizing dosages, and conducting long-term toxicity and individual difference studies to enhance the reliability and clinical applicability of the findings.

### 7.7 Other pharmacological effects

Members of the genus *Paris* possess a variety of pharmacological activities. In addition to those mentioned above, there are other activities as well. The research results of Sha et al. (Sha et al., 2024) indicate that PPPm-1 can improve offspring’s learning and memory abilities in aged pregnant rats. The mechanism of action is that PPPm-1 can effectively activate the Wnt/*β*-catenin signaling pathway, promote the expression of Wnt/1, *β*-catenin, CyclinD1, TCF-4 mRNA, and proteins, and inhibit the expression of GSK-3β mRNA and proteins, thereby enhancing the learning and memory capabilities of the offspring mice.

Acute myeloid leukemia (AML) is a category of malignant tumors originating from hematopoietic stem cells, characterized by diverse etiologies, strong heterogeneity of the disease, poor prognosis, and a relatively low long-term survival rate (Bai et al., 2019). Polyphyllin I (PPⅠ) can potentially become an effective therapeutic agent for treating human acute myeloid leukemia. PPⅠ triggers apoptosis in THP-1 and NB4 cells by reducing the levels of Bcl-2 while increasing the expression of Bax, cleaved caspase-3, and phosphorylated JNK. Additionally, treatment with PPⅠ leads to an increase in the expression levels of LC3-II and Beclin-1, and MDC staining reveals an increase in the number of autophagic vacuoles, indicating that PPⅠ promotes the process of autophagy. The mechanism is associated with inhibiting the AKT-mTOR signaling pathway (Tian et al., 2019).

Lung cancer is considered the leading cause of cancer-related deaths worldwide. A significant obstacle in the treatment of lung cancer is the emergence of resistance to osimertinib. Polyphyllin Ⅰ may become a drug capable of reversing osimertinib resistance. Polyphyllin I, isolated from the natural herb members of the genus *Paris*, possesses anti-cancer activity. The results indicate that Polyphyllin I may downregulate the PI3K/Akt signaling pathway and increase the expression of apoptosis-related proteins, thereby promoting cell apoptosis (Lai et al., 2021).

Diabetes is a chronic metabolic disease characterized by persistent hyperglycemia. It poses a long-term threat to health, and its complications affect multiple organs and tissues throughout the body. With economic growth and lifestyle changes, the prevalence of diabetes is continuously increasing, and currently, there is no cure for diabetes in clinical practice (Huang et al., 2024). Medicinal plants, as a treatment for diabetes, are accepted worldwide due to their long-term effectiveness and fewer side effects. The plants of *Paris* species can be used as a drug for the treatment of diabetes.

The experiment used a diabetic rat model induced by streptozotocin (STZ) and treated with *P. polyphylla* enriched with diosgenin (PPED) as the therapeutic drug. In the experiment, diabetic rats were intragastrically administered PPED at 200 mg/kg body weight (PPED-1) and 400 mg/kg body weight (PPED-2) for 28 consecutive days, with fasting blood glucose and body weight monitored every 7 days. The results showed that, compared to the diabetic group, the PPED treatment groups significantly reduced fasting blood glucose levels and promoted weight recovery. With the increase in PPED dosage, antioxidant indicators in the liver and kidneys were also improved. In addition, the treatment groups showed a reduction in glycated hemoglobin (HbA1c) and blood lipid levels, as well as improvements in protein, liver, and kidney function parameters. Most importantly, PPED treatment also improved the morphology of the islets and the granulation of *β*-cells (Kshetrimayum et al., 2023).

The pharmacological activity research of *Paris* species involves multiple diseases, demonstrating broad activity and clear mechanisms. However, there are limitations, such as the complex composition of extracts, the potential impact of high drug concentrations on result accuracy, the lack of long-term toxicity and tolerance studies, insufficient consideration of individual differences, and limited research on resistance mechanisms. Therefore, future studies should fully address these issues to enhance scientific rigor and clinical application value.

## 8 Toxicology

Members of the genus *Paris* is one of the main components of traditional Chinese patent medicines such as “Yunnan Baiyao” and “Jidesheng sheyao tablets,” which are widely used in clinical practice and have significant therapeutic effects. The “Pharmacopoeia of the People’s Republic of China” records that *Paris* species has slight toxicity. Research has found that in traditional clinical settings, excessive intake of RPS is linked to a range of side effects, spanning from gastrointestinal issues such as nausea, vomiting, and diarrhea, as well as gastric discomfort, to more serious symptoms like heart palpitations and convulsions (Liu et al., 2012).In recent years, with the increasing awareness of the safety of traditional Chinese medicine among people, scholars have also used cell experiments to successfully verify the hepatotoxicity of the *Paris* species (Li et al., 2023). However, the specific mechanism has not yet been clarified Future research should integrate clinical data to further validate the dose-dependency and individual differences of *Paris* species’s toxic effects, thereby providing a more scientific basis for its safety evaluation and clinical application.

*Paris* spp. have hepatotoxicity, with a mouse LD_50_ of 2.65 g/kg. In the subacute toxicity test, rats were given 0.53 g/kg of *Paris* spp. steroidal saponins daily, which resulted in sustained weight loss, decreased appetite, diarrhea, loose hair, difficulty breathing, abdominal distension, and ultimately death. After 2 weeks of continuous oral administration, scattered necrosis appeared in the liver tissue, the volume of surrounding liver cells increased, and no significant pathological changes were observed in the kidney tissue. **Folk detoxification** therapy is to boil water with 50 parts licorice, add 2 liang of white rice vinegar and ginger juice, half rinse and half take orally (University, 1976).

Zebrafish treated with PPⅥ and PⅦ showed hepatotoxic changes like liver phenotype alterations, hepatocyte issues, and biochemical index disorders, as found by Li et al. This study provides the first direct evidence of the hepatotoxicity of PPⅥ and PⅦ in a zebrafish *in vivo* model, which is associated with steroid biosynthesis. Lovastatin protects against this hepatotoxicity by regulating cholesterol metabolism and improving liver structure and gene expression in zebrafish (Li et al., 2022).

After weighing, six-week-old male rats were divided into a normal control group and an RPS group. The latter was administered 200 mg/kg of *Rhizoma Paridis* saponins (1/8 LD_50_) orally every day for 45 days. During the experiment, the body weight of each rat was measured weekly, and urine samples were collected. The study demonstrated that RPS induces mild liver injury (elevated serum AST, AKP, ALT, and *γ*-GT, with hepatic tissue lesions), oxidative stress (increased ROS, MDA, and 8-OHdG), and inflammatory responses (upregulation of COX-2, IL-1*β*, and NF-*κ*B). These abnormalities were alleviated by co-administration with curcumin (Man et al., 2016). This research elucidates the toxicity mechanisms of RPS and provides a scientific basis for the development of safe medicinal applications.

However, research on the neurotoxicity of *Paris* spp. is currently relatively limited. RPS at doses of 250 and 500 mg/kg can shorten the sleep latency and prolong the sleep duration in mice, exhibiting a synergistic effect with sodium pentobarbital, although its efficacy is weaker than that of estazolam. Meanwhile, RPS at doses of 100, 250, and 500 mg/kg does not significantly affect the motor coordination in mice (Liu et al., 2012). This indicates that RPS possesses sedative and hypnotic activities without impacting motor coordination. This experiment provides a scientific basis for its application in traditional medicine.

*Paris* species is the main components of traditional Chinese medicines such as “Yunnan Baiyao” and have significant therapeutic effects. Cell experiments and zebrafish models have revealed partial toxicity mechanisms, providing evidence for the safety research of traditional Chinese medicine. However, the toxicity mechanisms have not been fully elucidated, clinical toxicity data are limited, traditional detoxification methods lack scientific validation, and individual differences have not been adequately considered in the studies. In the future, personalized toxicity assessment and detoxification protocols should be developed to enhance the safety and clinical application value of *Paris* species.

## 9 Practical applications

Botanical drugs, which use parts or whole plants for therapeutic purposes, are widely popular around the world. These botanical drugs offer a rich resource for new drug and health product development and are gaining research interest due to their lower toxicity, fewer side effects, and unique benefits for treating incurable, chronic, and geriatric diseases (Yu et al., 2012). Complementary and alternative medicine (CAM), is increasingly valued in cancer treatment for its ability to inhibit tumor growth, enhance immunity, and augment the efficacy of chemotherapy, radiotherapy, targeted, and immunotherapies, while also alleviating treatment side effects (Wang et al., 2020).

*Paris* spp., known for its medicinal properties, is widely used to treat conditions like mumps, bites, stomach issues, joint pain, mastitis, and burns. Despite gaps in pharmacological evidence, its low toxicity and unique effects on chronic and geriatric diseases make it a valuable resource for drug development. However, the market application rate of *Paris* species-based patents and products is low (Li, 2022). Members of the genus *Paris* are utilized in various formulations, have obtained some patents, and extracts like *P. polyphylla* are used in personal care products.

In pharmaceutical research and development, *Paris* species have become an indispensable ingredient in many medicines due to their significant medicinal value. Yunnan Baiyao (approval number: Z53020798), a blend of traditional Chinese medicine and modern practice, contains key ingredients like *P. polyphylla* and is known for its therapeutic effects on pain and bleeding across various medical fields. It has expanded into new dosage forms and has been recognized for its oral health benefits with toothpaste, all while being classified as a state confidential formula.

Gongxue Ning Capsules (approval number: Z20020087), with *P. polyphylla* var. yunnanensis as the main ingredient, effectively treats various uterine bleedings in gynecology, while Jidesheng Sheyao Tablets (approval number: Z32020048) are well-regarded for treating snake and insect bites. Re Du Qing Pian (approval number: Z53020800), primarily containing members of the genus *Paris*, is used for clearing heat, detoxifying, reducing swelling, and dispersing lumps, addressing symptoms like mumps and upper respiratory tract infections caused by heat toxicity (Zhang and Du, 2021).

Yunnan Baiyao Group has expanded the use of *Paris* species into botanical skincare, showcasing its potential in beauty and skincare, and Winona, as a medical skincare brand, uses natural ingredients like Tattoo Fruit Oil, Portulaca Oleracea Extract, Camellia Oil, and *P. polyphylla* Smith Extract to effectively treat skin conditions such as eczema, psoriasis, cheilitis, and allergic dermatitis (Zuo et al., 2015). In India, it is used to treat burns, cuts, diarrhea, dysentery, fever, stomachaches, and wounds (Pfoze et al., 2013; Tariq, 2016). Not only that, but the rhizome of this plant also holds significant medicinal value in Nepal, traditionally used to treat snake and insect bites, mitigate the toxic effects of narcotics, and address issues such as wounds, fever, and food poisoning (Kunwar et al., 2020).

**FIGURE 6:**
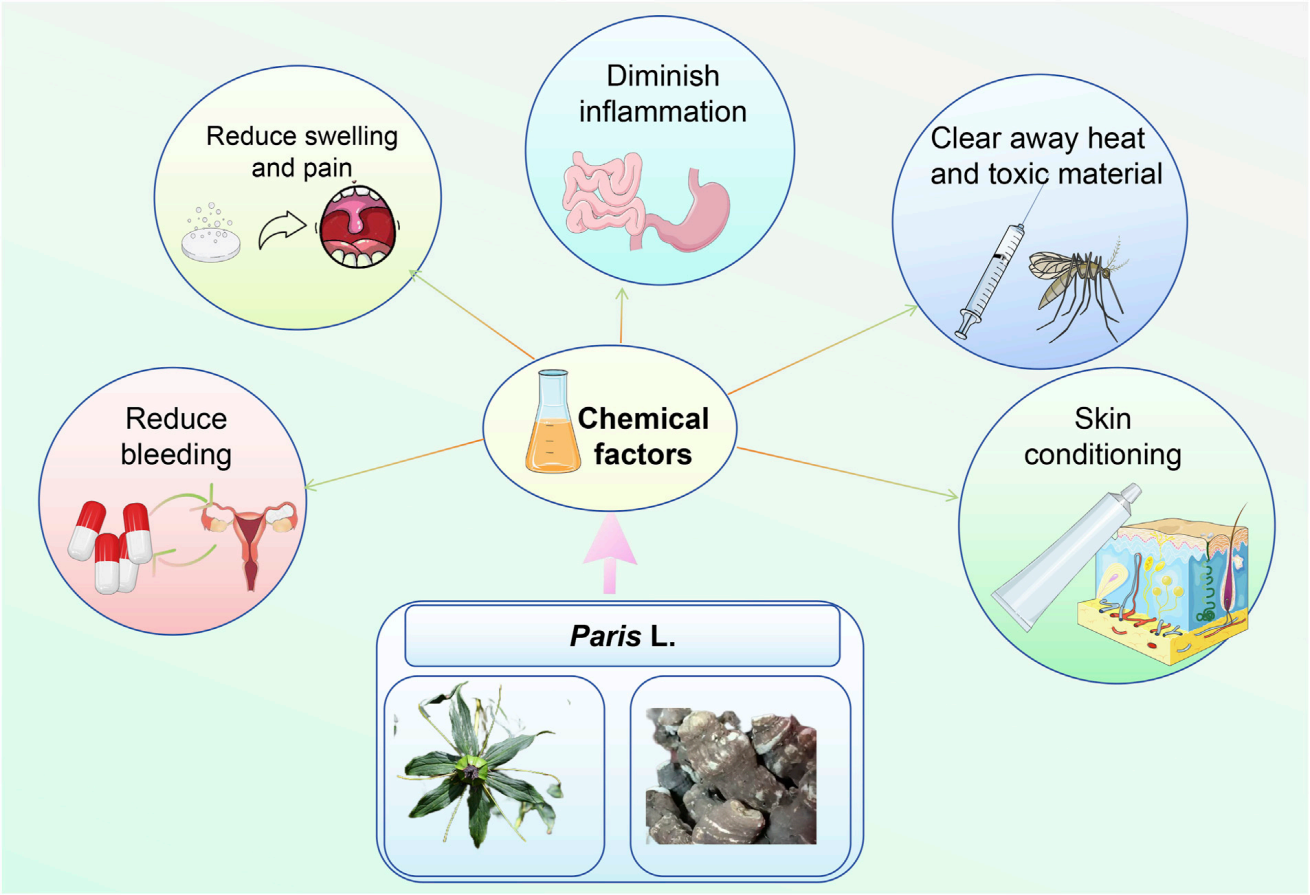
The practical applications of *Paris* spp.

It is evident that *Paris* spp. possesses significant medicinal value, which can be maximized by thoughtfully blending and proportioning it with various traditional Chinese medicinal herbs to formulate targeted remedies for enhanced therapeutic outcomes. The practical applications of *Paris* species are detailed in Figure 6. Despite the potential of *Paris* spp. in cancer treatment and complementary medicine, challenges persist due to insufficient pharmacological evidence and limited clinical data. In addition, research on the neurotoxicity of *Paris* spp. is currently relatively limited. Future work should prioritize increasing research support, validating traditional uses via modern studies, and boosting market competitiveness to maximize the potential of *Paris* species in both healthcare and commercial settings.

## 10 Discussions and Prospects

This article reviews the latest research progress of *Paris* spp. in terms of botanical characteristics, chemical components, ethnic pharmacology, pharmacological effects, and practical applications. The rhizome of *P. polyphylla* is the main source of their medicinal value, and studies have shown that more than four hundred different metabolites have been successfully isolated from members of the genus *Paris*. These metabolites mainly include steroidal saponins, flavonoids, phytosterols, triterpenoids, insect allergens, etc., and their discovery provides important reference indicators for assessing the quality of medicinal materials of *Paris* species. Modern pharmacological experiments have further confirmed the various pharmacological effects of *Paris* species, including anticancer, hemostatic, antitumor, anti-inflammatory analgesic, and antibacterial effects. These research results provide scientific validation for the traditional use of members of the genus *Paris* and lay a solid foundation for their application in modern medicine.

This rhizomatous herb is found in Bangladesh, Bhutan, China, India, Laos, Myanmar, Nepal, Taiwan, Thailand, and Vietnam (Paul et al., 2015)*. Paris* species has different traditional uses in different regions. In China,the rhizome of *Paris* species (Chong Lou) is used as an integral part of treatments for conditions such as abnormal uterine bleeding, cancer, snake bites, and skin diseases (Qin et al., 2018b). Rhizome Paridis, a key ingredient in “Yunnan Baiyao” and “Gong Xue Ning” capsules, is also found in many other Chinese patented medicines. For instance, it is included in “Lou Lianjiaonang” and “Jinfukang Koufuye,” which serve as adjuvant therapies to enhance the benefits of cancer treatment (Ding et al., 2021). In addition to having numerous uses in China, *Paris* spp. also has different applications in other countries. In Nepal, *Paris* species’ rhizome juice relieves gastric and menstrual pain, and its paste treats cuts, wounds, deworms, and helps treat fever, diarrhea, dysentery, and acts as an antidote (Malla et al., 2015; Pfoze et al., 2013).In India, the raw rhizomes of *Paris* species are consumed to treat stomach ulcers, while its leaves and rhizome are used for treating diabetes, stomach worms, and as a tonic (Kshetrimayum et al., 2023; Phurailatpam et al., 2022). From the above statements, it is evident that *Paris* species possesses significant medicinal value.

In addition to the rhizome, chemical components with specific pharmacological effects can also be extracted from other parts. The fruit peel and leaves of members of the genus *Paris* also contain various metabolites, mainly steroidal saponins, C-21 steroids, insect allergens, and flavonoids (Su, 2023; Zhang, 2022). Polyphyllin V, isolated from the stem and leaves of *P. polyphyl*la var. *chinensis*, affects the cell cycle distribution in MDA-MB-231 cells and induces G2/M phase arrest (Qin et al., 2020). Not only that, but the flavonoid compounds isolated from the stem and leaves of *P. polyphylla* var. *chinensis* also has anti-platelet aggregation effects (Zhang et al., 2023b). The above content demonstrates the potential value of the non-medicinal parts of *Paris* species. Experimental results reveal their diverse pharmacological activities, providing scientific evidence for the development potential of these non-medicinal parts and offering strong support for the comprehensive utilization of plant resources.

The growth cycle of *Paris* speces is relatively long, which leads to a gradual decrease in their population numbers in the natural world, thereby driving up their market prices. Therefore, the phenomenon of counterfeiting in botanical medicines occurs. However, researching the metabolites from non-rhizome parts of *Paris* species can improve the entire plant’s utilization efficiency, achieve sustainable resource development, and reduce dependence on wild populations. Delving into the chemical components, pharmacological effects, and resource utilization models of these non-medicinal parts has opened up new ideas and pathways for the industrial development of members of the genus *Paris*. These research advancements are expected to promote the efficient use of *Paris* spp. and potentially drive their broader application in pharmaceutical and other related industries.

*Paris* species’ metabolites pennogenyl saponins (PHAC-A) and dioscins (PHAC-B), which have various pharmacological effects, were isolated from the rhizomes of *P. polyphylla* Smith. The experiment used mice for *in vitro* tests to evaluate the inhibitory effects of the extracts on sperm motility and viability. The results showed that, compared with gossypol acetic acid (positive control), PHACs had better inhibitory effects on sperm activity and survival rate. Meanwhile, PHAC-B exhibited stronger inhibitory activity than PHAC-A, and its activity was directly proportional to the dose. The results suggest that they have the potential to serve as effective contraceptive agents (Shen et al., 2010). Literature suggests that members of the genus *Paris*, particularly Polyphyllin I, may protect skin and treat diseases by enhancing SIRT3 activity, which deacetylates and activates SOD2, reducing oxidative stress and apoptosis in HaCaT cells exposed to UVB radiation (Gao et al., 2023).The studies have respectively revealed the potential of *Paris* species metabolites in contraception and skin protection. However, further validation through *in vivo* experiments, toxicity assessment, and mechanistic exploration is needed to confirm their safety and efficacy, laying the foundation for clinical application.

As experimental research on members of the genus *Paris* continues to deepen, our understanding of their pharmacological effects becomes increasingly profound, which aids in expanding their application in clinical medicine. The research achievements in plant chemistry and pharmacology provide a solid theoretical foundation for in-depth study and development of the medicinal value of *Paris* species. These discoveries may promote the development of new drugs targeting complex diseases such as cancer and diabetes, bringing more far-reaching impacts and contributions to human health.

Although research on *Paris* spp. has achieved remarkable results, there are still many areas that urgently need further in-depth exploration. With the continuous decline of its wild population resources, it is imperative to conduct further research to develop effective conservation measures and sustainable utilization strategies, thereby reducing the dependence on wild populations. Moreover, establishing standardized methods and quality control standards for *Paris* spp. and their products is particularly important, which will help ensure the consistency and reliability of product quality. Meanwhile, actively assessing the potential toxicity of *Paris* spp. and its components is of vital importance to ensure their safe application in medicine, food, cosmetics, and health products.

For the known pharmacological effects of *Paris* spp., such as anti-tumor, anti-inflammatory, and antibacterial activities, an in-depth study of their mechanisms of action is crucial to ensure their safety and efficacy in traditional medicine. Although *Paris* spp. are widely used in many countries, their pharmacological and toxicological effects on patients with underlying diseases or special populations, such as the elderly, children, pregnant women, and nursing mothers, are still unclear, requiring further research and evaluation. Therefore, there are still many issues worth exploring in the field of research on *Paris* spp. We need ongoing research efforts to address these challenging issues to promote the broader application of *Paris* spp. in modern and traditional medicine.

In summary, *Paris* spp. has demonstrated outstanding potential for application in various fields and has proven their significant therapeutic effects in clinical practice, which further highlights the necessity for in-depth research on members of the genus *Paris*. We hope that by further exploring their intrinsic value, we can open up new horizons and research directions for future work, expecting to achieve more scientific breakthroughs and practical applications.

## Glossary

WHO: World Health Organization
GBIF: Global Biodiversity Information Facility
Glu: Glucose
Rha: Rhamnose
Xyl: Xylose
Ara: Arabinose
Gal: Galactose
Fuc: Fucose
Api: Apiose
P53: Tumor Protein p53
MCF-7 cell: *Michigan Cancer Foundation - 7*
MIC: *Minimum Inhibitory Concentration*
IC50: *Half-maximal Inhibitory Concentration*
HepG2 cell: Human Hepatocellular Liver Carcinoma cell
HEK293 cell: Human Embryonic Kidney 293 cells
JNK: C-Jun N-terminal Kinase
Bcl-2: **B-cell CLL/lymphoma 2**
C-BSA: Cationized Bovine Serum Albumin
FIB: The Level of Fibrinogen
PPED: *Paris polyphylla* Sm. Extract Enriched
SOD2: Superoxide Dismutase 2
TSSRs: Total Steroidal Saponins of the Rhizomes
LD50: Median Lethal Dose
RPS: Rhizoma Paridis Saponins
ECA 100: Eca-109 Human Csophageal cells
NF-*κ*B: Nuclear Factor kappa B
Drp 1: Dynamin-related Protein 1
PDK: Pyruvate Dehydrogenase Kinase Isozyme 1
mTOR: Mammalian Target of Rapamycin
VEGF: *Vascular Endothelial Growth Factor*
MCT 4: Monocarboxylate Transporter 4
cyt-c: Cytochrome C
LPS: *Lipopolysaccharide*
NO: Nitric Oxide
PEG 2: Prostaglandin E2
*RAW* 264.7 cells: *Mononuclear MacrophageleukemiaRAW* 264.7 cells
iNOS: Inducible Nitric Oxide Synthase
COX-2: Cyclooxygenase-2
MMP-9: Matrix Metalloproteinase-9
MN: Membranous Nephropathy
AML: Acute Myeloid Leukemia
PPI: Polyphyllin I
STZ: Streptozotocin
CAM: Complementary and Alternative Medicine
TSSAPs: Total Steroidal Saponins of the Above-ground Parts
PPED: *Paris polyphylla* Enriched with Diosgenin
ACLT: Anterior Cruciate Ligament Transection
SAP: Severe Acute Pancreatitis

## References

[B1] Bai H. Wen J. Wang D. Zhang W. (2019). Progress of dendritie cell vaccine in immunotherapy of acute myeloid leukemia. Med. Recapitulate. 25 (10), 1900–1904. Available online at: https://link.cnki.net/urlid/11.3553.R.20190510.1322.012.

[B2] Bureau, (1990). Deyang medicine collection. Dehong Ethnic Publishing House. Available online at: http://book.duxiu.com/bookDetail.jsp?dxNumber=000004094574&d=1D7108E967DE7197D9831313E9DE10A3&fenlei=160517.

[B3] Chang Y. (1996). Anti-cancer traditional Chinese medicine. Hunan Science and Technology Press. Available online at http://book.duxiu.com/bookDetail.jsp?dxNumber=000000516917&d=DD728247A5ACFD82E4C0F9B580A81D53&fenlei=16051705.

[B4] Che C. T. Wang Z. J. Chow M. S. Lam C. W. (2013). Herb-herb combination for therapeutic enhancement and advancement: theory, practice and future perspectives. Molecules 18 (5), 5125–5141. 10.3390/molecules18055125 23644978 PMC6269890

[B5] Chen M. Jiang H. (2017). Pharmacology and clinical analysis of commonly used traditional Chinese medicine for skin diseases. 2nd Edition. China, China Science and Technology Press. Available online at http://book.duxiu.com/bookDetail.jsp?dxNumber=000017286425&d=CD4E4D80F4E0876CFFD1C635BDA40A79&fenlei=160512.

[B6] Chen M. Ye K. Zhang B. Xin Q. Li P. Kong A. (2019). Paris Saponin II inhibits colorectal carcinogenesis by regulating mitochondrial fission and NF-κB pathway. Pharmacol. Res. 139, 273–285. 10.1016/j.phrs.2018.11.029 30471409 PMC8108001

[B7] Chen Q. Yan S. (2012). Research progress on pharmacological effects and toxic reactions of *Paris* . Her. Med. 31 (07), 886–888. Available online at: https://kns.cnki.net/kcms2/article/abstract?v=bnAgTRNiloJpci4ZsvweMWQwkJ_SGDn0NMHPx8t26U-Hfjzy4TkNHuB9_SsAFiJpGsG0C6wgfANc88jBRhRCg7WW4M3EjGNpeGOTT-wBojaSTqoVZyQqsmJQDZVQ1c3Ebk4MUJyQ9mTg36RIDwSD8A==&uniplatform=NZKPT&language=CHS.

[B8] Cheng G. Xue Y. Fang F. Sun G. Lu Y. Ji Y. (2021). Promotion of Ros-mediated Bax/Cyt-c apoptosis by polyphyllin II leads to suppress growth and aggression of glioma cells. Transl. Cancer Res. 10 (9), 3894–3905. 10.21037/tcr-21-966 35116689 PMC8797898

[B9] Cheng H. Jiao L. Su J. Jing C. Meng W. Qinglin Li (2024). Mechanism of Paris polyphylla saponin Ⅱ inducing autophagic to inhibit angiogenesis of cervical cancer. Naunyn-Schmiedeberg's Archives Pharmacol. 10.1007/s00210-023-02794-x 37906274

[B10] Cheng J. Chen W. (2016). Hakka Chinese herbal atlas and folk formula testing. The Medicine Science and Technology Press of China. Available online at http://book.duxiu.com/bookDetail.jsp?dxNumber=000016146982&d=0A568CA4F0059FA62B4ED8DC3E5B964B&fenlei=1605160232.

[B11] Cui J. Tang Li (2007). Introduction to traditional medicine of Chinese ethnic minorities. Central University for Nationalities Press.

[B12] Cui Y. (2006). Study on the active chemical constituents ofparis polyphylla smith: isolation andstructure characterization. Beijing; Beijing University Of Chemical Technology, 74. Available online at https://kns.cnki.net/kcms2/article/abstract?v=n93avYlexq8XKXHIqxviuxQMe7bV2P94J1QqjO_3RepC6iF3afVqlR8j8gMdWtDyeAzWh2WwCHCFOM1JEF-bumplOpAt0etksB4y3FZoQny9tQ4V5fwpugymeWnCAz627pyX4rAMH14IX86H2MdHfwa6-4VZvAH_&uniplatform=NZKPT&language=CHS.

[B13] Dai H. Mei W. (2008). Li ethnic medicine chronicles, Vol. 1. China, China Science and Technology Press. Available online at http://book.duxiu.com/bookDetail.jsp?dxNumber=000007418886&d=F8C5EC1B19444B963C5AE88CB495E26F&fenlei=1605160103.

[B14] Ding L. Zhao M. Li Y. Chen L. Wang Z. Wang Z. (2018). Study on the anti-nociceptive and anti-inflammatory effects of the extract of aerial part and rhizome of *Paris* polyphylla var. chinensis. Nat. Prod. Res. Dev. 30 (05), 832–839. 10.16333/j.1001-6880.2018.5.017

[B15] Ding Y. Zhao Y. Zhang J. Zuo Z. Zhang Q. Wang Y. (2021). The traditional uses, phytochemistry, and pharmacological properties of *Paris* L. (Liliaceae): a review. J. Ethnopharmacol. 278, 114293. 10.1016/j.jep.2021.114293 34102270

[B16] Ding Y. G. Zhao Y. L. Zhang J. Zuo Z. T. Zhang Q. Z. Wang Y. Z. (2021). The traditional uses, phytochemistry, and pharmacological properties of *Paris* L. (Liliaceae): a review. J. Ethnopharmacol. 278, 114293. 10.1016/j.jep.2021.114293 34102270

[B17] Du Z. Zhang G. Sun J. (2024). Research progress on anti-tumor effect and mechanism of saponins in Chonglou. Yunnan Med. 45 (02), 73–75. Available online at: https://kns.cnki.net/kcms2/article/abstract?v=f1ZyUc11mdpLaUcyqASuu81CcVE8uc4zWs81JDFJnmlCxFrQuL33UerltRdaY1AeJruaFBEj9PGXKo16yLDg8sZIll6R0_yP4mf6YjXJKpRdNxjSdzfo-YJqsBgP0Y9_MMpSFS7ai0sT9anWlXT8Fw==&uniplatform=NZKPT&language=CHS.

[B18] Duan X. Yue M. Yang J. Bai X. Lu J. Li H. (2023). Chemical constituents from *Paris* rugosa rhizomes and their antimicrobial activities. China J. Chin. Materia Medica 48 (11), 2981–2988. 10.19540/j.cnki.cjcmm.20230122.201 37381958

[B19] Duyen N. T. Vinh L. B. Phong N. V. Khoi N. M. Ha D. T. Long P. Q. (2022). Steroid glycosides isolated from *Paris* polyphylla var. chinensis aerial parts and paris saponin II induces G1/S-phase MCF-7 cell cycle arrest. Carbohydr. Res. 519, 108613. 10.1016/j.carres.2022.108613 35752103

[B20] Fang M. (2014). De'ang ethnic medicine collection. Dehong Ethnic Publishing House. Available online at http://book.duxiu.com/bookDetail.jsp?dxNumber=000015777003&d=75246D55D445504D745F90CF12619824&fenlei=160518.

[B21] Fang Z. Hui Z. Jinghua Z. (2007). The medicine science and technology press of China. Tujia medicine chronicles last of two or three volumes.

[B22] Gao Z. Wang D. Wu S. Huang Y. Man J. Gao R. (2023). Study on the protective effect of polyphyllin Ⅰ on HaCat cells iniured by ultraviolet ray. J. Yunnan Agric. Univ. Nat. Sci. 38 (04), 621–626. Available online at: https://link.cnki.net/urlid/53.1044.s.20230905.1020.

[B23] Gu J. Yang T. Yang M. Zhan Z. Zhang J. (2023). Herbal textual research on paridis rhizoma in famous classical formulas. Chin. J. Exp. Traditional Med. Formulae. 10.13422/j.cnki.syfjx.20240167

[B24] Guan L. Zheng Z. Guo Z. Xiao S. Liu T. Chen L. (2024). Steroidal saponins from rhizome of *Paris* polyphylla var. chinensis and their anti-inflammatory, cytotoxic effects. Phytochemistry 219, 113994. 10.1016/j.phytochem.2024.113994 38244959

[B25] Guo K. Ren X. Mu R. Zhou T. Li D. Hu H. (2021). Ecdysteroids and spirosterane steroids from the traditional Chinese medicine *Paris* polyphylla var. yunnanensis. Phytochem. Lett. 45, 117–120. 10.1016/j.phytol.2021.08.008

[B26] Guo Y. Liu Z. Li K. Cao G. Sun C. Cheng G. (2018). *Paris* polyphylla-derived saponins inhibit growth of bladder cancer cells by inducing mutant P53 degradation while up-regulating CDKN1A expression. Curr. Urol. 11 (3), 131–138. 10.1159/000447207 29692692 PMC5903466

[B27] He H. Sun Y. P. Zheng L. Yue Z. G. (2015). Steroidal saponins from *Paris* polyphylla induce apoptotic cell death and autophagy in A549 human lung cancer cells. Asian Pac J. Cancer Prev. 16 (3), 1169–1173. 10.7314/apjcp.2015.16.3.1169 25735350

[B28] He H. Zheng L. Sun Y. P. Zhang G. W. Yue Z. G. (2014). Steroidal saponins from *Paris* polyphylla suppress adhesion, migration and invasion of human lung cancer A549 cells via down-regulating MMP-2 and MMP-9. Asian Pac J. Cancer Prev. 15 (24), 10911–10916. 10.7314/apjcp.2014.15.24.10911 25605200

[B29] He J. Chen G. Tang Y. Chang J. (2019). Effects of polyphyllin Ⅶ on pathological damage and extracellular matrix in osteoarthritis rats. Pharmacol. Clin. Chin. Materia Medica. 35 (06), 49–53. 10.1016/j.biopha.2019.109189

[B30] He J. Yu S. Guo C. Tan L. Song X. Wang M. (2019). Polyphyllin I induces autophagy and cell cycle arrest via inhibiting PDK1/Akt/mTOR signal and downregulating cyclin B1 in human gastric carcinoma HGC-27 cells. Biomed. Pharmacother. 117, 109189. 10.1016/j.biopha.2019.109189 31387191

[B31] He L. Yan X. Wen S. Zhong Z. Hou Z. Liu F. (2023). *Paris* polyphylla extract attenuates colitis in mice by regulating PPAR-gamma mediated Treg/Th17 balance. J. Ethnopharmacol. 314, 116621. 10.1016/j.jep.2023.116621 37164256

[B32] Hong F. Gu W. Jiang J. Liu X. Jiang H. (2019). Anticancer activity of polyphyllin I in nasopharyngeal carcinoma by modulation of lncRNA ROR and P53 signalling. J. Drug Target. 27 (7), 806–811. 10.1080/1061186X.2018.1561887 30601067

[B33] Hu J. (2022). Studies on antiglioma chemical constituents of *Paris* polyphylla var. stenophylla. Chinese People's Liberation Army Air Force Medical University, 103. 10.27002/d.cnki.gsjyu.2022.000225

[B34] Hu J. Lu Y. Zheng S. Tian Y. Li T. Tang H. (2023). Steroid and triterpenoid saponins from the rhizomes of *Paris* polyphylla var. stenophylla. Chin. J. Nat. Med. 21 (10), 789–800. 10.1016/S1875-5364(23)60486-8 37879796

[B35] Hua D. (2015). Studies on chemical constituents of *Paris* polyphylla var. latifolia. The fourth military medical university, 94. Available online at https://kns.cnki.net/kcms2/article/abstract?v=6RlcORkFSJS_hf5v_pyNoKfkulIWZlzCsvN0hZTjD7pxOIUjpGGnRLuU2DZPaOBpaQmSZpi0k9shuhkBJIBA7GCwYQqq-rpqlZwUhq34bVueir8ASkKV7nZfS7prJKSbDOlomJOlLeoroywJdNUqAvf8K1i_YTBD&uniplatform=NZKPT&language=CHS.

[B36] Huang G. Liu R. (2007). The protective effect of *paris* polyphylla on the kidneys of membranous nephropathy rats. Guangdong Med. J. (04), 527–529. 10.13820/j.cnki.gdyx.2007.04.011

[B37] Huang S. Gong H. Zhao L. Zhang Y. (2024). Risk factor analysis and path research of traditional Chinese medicine appropriate technology in diabetic patients. Hebei J. Traditional Chin. Med. 46 (05), 799–803. Available online at: https://kns.cnki.net/kcms2/article/abstract?v=bd42u7TqVAH--aQbaRxxrOmUDZeyOXZ_v7V5mUI-62aFOqI6uhrpdXElCIVvb7IUNK5LnwHMRypyWVGWvXEHqcWhqdC6lZsHsBrFXksxJCqwR9OcgRtBStcoc7z7upnOCAJnmdlITrLcTlcB3ZZGPg==&uniplatform=NZKPT&language=CHS.

[B38] Institute, Nujiang Lisu Autonomous Prefecture, Inspection, Yunnan Provincial Institute, Medicine, Yunnan University Of Traditional (2021). The medical plant atlas of the Lisu ethnic group in nujiang, 1. Yunnan University press. http://book.duxiu.com/bookDetail.jsp?dxNumber=000030527929&d=997D0BCFA33A7E7E18F323DE459A666A&fenlei=160518.

[B39] Institute Of Traditional Chinese Medicine, Chinese Academy Of Sciences (1960). Sichuan traditional Chinese medicine chronicles volume, 1. Sichuan People's Publishing House. Available online at http://book.duxiu.com/bookDetail.jsp?dxNumber=000030004086&d=7BC9ECEA54CB20DBC4E26087E43EF3B1&fenlei=1605160103.

[B40] Jiang B. (2017). Illustrated catalogue of medicinal plants of the Bai ethnic group. China Traditional Chinese Medicine Press. Available online at http://book.duxiu.com/bookDetail.jsp?dxNumber=000016628731&d=73647E2A30A9B6BFB20433EC82ECA34B&fenlei=17090704.

[B41] Jiang B. Xiao C. (2021). Modern research and application of medicinal plants with Bai ethnic characteristics. China Tradit. Chin. Med. Press. JiangXiao-691. Available online at: http://book.duxiu.com/bookDetail.jsp?dxNumber=000019321121&d=DCAFCBF0C58FCF14FECE6AAA3349A810&fenlei=17090704.

[B42] Jiang X. He Y. Hou X. Yang B. Wang L. Li F. (2022). Two new polyhydroxylated steroidal glycosides from *Paris* polyphylla var. yunnanensis. Phytochem. Lett. 49, 171–176. 10.1016/j.phytol.2022.03.006

[B43] Jing S. Wang Y. Li X. Man S. Gao W. (2017). Chemical constituents and antitumor activity from *Paris* polyphylla Smith var. yunnanensis. Nat. Prod. Res. 31 (6), 660–666. 10.1080/14786419.2016.1219861 27687140

[B44] Kshetrimayum V. Chanu K. D. Ghosh S. Haldar P. K. Mukherjee P. K. Sharma N. (2023). *Paris* polyphylla Sm. extract enriched with diosgenin as an antidiabetic agent: *in vitro* and *in vivo* study. Phytomedicine Plus Int. J. phytotherapy Phytopharm. 3 (4), 100497. 10.1016/j.phyplu.2023.100497

[B45] Kshetrimayum V. Heisnam R. Keithellakpam O. S. Radhakrishnanand P. Akula S. J. Mukherjee P. K. (2023). *Paris* polyphylla Sm. Induces reactive oxygen species and caspase 3-mediated apoptosis in colorectal cancer cells *in vitro* and potentiates the therapeutic significance of fluorouracil and cisplatin. Plants 12 (7), 1446. 10.3390/plants12071446 37050072 PMC10097216

[B46] Kunwar R. M. Adhikari Y. P. Sharma H. P. Rimal B. Jentsch A. Charmakar S. (2020). Distribution, use, trade and conservation of *Paris* polyphylla Sm. in Nepal. Glob. Ecol. Conserv. 23, e01081. 10.1016/j.gecco.2020.e01081

[B47] Lai L. Shen Q. Wang Y. Chen L. Lai J. Wu Z. (2021). Polyphyllin I reverses the resistance of osimertinib in non-small cell lung cancer cell through regulation of PI3K/Akt signaling. Toxicol. Appl. Pharmacol. 419, 115518. 10.1016/j.taap.2021.115518 33812963

[B48] Li F. R. Jiao P. Yao S. T. Sang H. Qin S. C. Zhang W. (2012). *Paris* polyphylla Smith extract induces apoptosis and activates cancer suppressor gene connexin 26 expression. Asian Pac J. Cancer Prev. 13 (1), 205–209. 10.7314/apjcp.2012.13.1.205 22502669

[B49] Li J. Wu Z. Chen G. Wang X. Zhu X. Zhang Y. (2023). Formosanin C inhibits non-small-cell lung cancer progression by blocking MCT4/CD147-mediated lactate export. Phytomedicine Stuttg. 109, 154618. 10.1016/j.phymed.2022.154618 36610137

[B50] Li L. Zhang J. Cheng W. Di F. Wang C. An Q. (2024). Saponins of *Paris* polyphylla for the improvement of acne: anti-inflammatory, antibacterial, antioxidant and immunomodulatory effects. Molecules 29 (8), 1793. 10.3390/molecules29081793 38675613 PMC11052371

[B51] Li P. Fu J. Wang J. Ren J. Liu J. (2011). Extract of *Paris* polyphylla simth protects cardiomyocytes from anoxia-reoxia injury through inhibition of calcium overload. Chin. J. Integr. Med. 17 (4), 283–289. 10.1007/s11655-011-0704-4 21509672

[B52] Li R. (2022). Research on key traditional Chinese medicine industry in yunnan. Kunming: Yunnan Science and Technology Press, 213.

[B53] Li T. Nong T. Qin S. Qin X. (2005). Practical Yao medicine. The Medicine Science and Technology Press of China. Available online at http://book.duxiu.com/bookDetail.jsp?dxNumber=000005127973&d=FA4803721CD4DB948B1B8204A5CE4FAE&fenlei=160518.

[B54] Li W. (2023). Overview of extraction methods for flavonoids. Famm Prod. Process. 14, 75–78. 10.27002/d.cnki.gsjyu.2023.000119

[B55] Li X. Liu Y. Liao S. Lin C. Moro A. Liu J. (2021). Polyphyllin VII induces apoptosis and autophagy via mediating H2O2 levels and the JNK pathway in human osteosarcoma U2OS cells. Oncol. Rep. 45 (1), 180–190. 10.3892/or.2020.7866 33416129 PMC7709821

[B56] Li Z. (2011). Research and application of toxic drugs in ethnic minority areas of China. Central University for Nationalities Press.

[B57] Li Z. Chen M. Guo S. Fan Q. Lin Z. Zhong X. (2023). Hepatotoxicity mechanism of paridis rhizoma based on zebrafish model combined with network pharmacology. World Chin. Med. 18 (06), 739–747+755. 10.1016/j.fitote.2023.105498

[B58] Li Z. Li J. Huang X. Meng F. (2021). Inhibition of Wnt/β - catenin signaling by *paris* polyphylla polyphyllin Ⅶ reduces interleukin-1 β - induced apoptosis of articular chondrocytes and secretion of inflammatory mediators. Chin. J. Gerontology 41 (21), 4815–4819.

[B59] Li Z. Tang Y. Liu Z. Fan Q. Chen M. Lin Z. (2022). Hepatotoxicity induced by PPⅥ and PPⅦ in zebrafish were related to the Cholesterol disorder. Phytomedicine Stuttg. 95, 153787. 10.1016/j.phymed.2021.153787 34782205

[B60] Liu G. Wang Z. Li T. Du J. Du X. Cao X. (2015). Effect of polysaccharides from the leaves of *Paris* polyphylla on immune function and antioxidant capacities of mouse spleen tissue in a D-galactose-induced aging mouse model. Sci. Technol. Food Industry 36 (16), 366–369+383.

[B61] Liu J. Liu Y. Li H. Wei C. Mao A. Liu W. (2022). Polyphyllin D induces apoptosis and protective autophagy in breast cancer cells through JNK1-Bcl-2 pathway. J. Ethnopharmacol. 282, 114591. 10.1016/j.jep.2021.114591 34481873

[B62] Liu X. Wang L. Long Y. Sun L. Wang Q. (2014). Chemical constituents from *Paris* mairei. China J. Chin. Materia Medica 39 (16), 3107–3111. 10.4268/cjcmm20141620 25509296

[B63] Liu Y. (2018). Studies on chemical constituents of *Paris* vietnamensis and *Paris* mairei. Chinese People's Liberation Army Air Force Medical University, 312. Available online at https://kns.cnki.net/kcms2/article/abstract?v=6RlcORkFSJRbYnYhPckGSWIo_5lBMiddMcBTDIuzCOMNAZC5hzx4jXXQYyzM0Gso8MmBQAZaQxYcvZgNXr1D8N_B-f2njzW0eqIjxTdWKK1D9JoVydyE3EquXvyqkpqUfAfIpHUndLh2qEgQPsN-zmTxRkVcRqxNggAWUlrgNlI=&uniplatform=NZKPT&language=CHS.

[B64] Liu Y. Liu M. Bi L. Tian Y. Qiu P. Qian X. (2023). Cytotoxic steroidal glycosides from the rhizomes of *Paris* polyphylla var. yunnanensis. Phytochem. Oxf. 207, 113577. 10.1016/j.phytochem.2022.113577 36587887

[B65] Liu Y. Tang N. Qian X. Y. Li T. Y. Zhang Q. Qiu P. C. (2023). New steroidal saponins from the roots of *Paris* verticillata. Nat. Prod. Res. 38 (15), 2688–2696. 10.1080/14786419.2023.2200184 37067218

[B66] Liu Y. Tian X. Hua D. Cheng G. Wang K. Zhang L. (2016). New steroidal saponins from the rhizomes of *Paris* delavayi and their cytotoxicity. Fitoterapia 111, 130–137. 10.1016/j.fitote.2016.04.018 27118322

[B67] Liu Y. Xu F. Fan M. Duan B. (2019). Application of *Paris* (Melanthiaceae) in Chinese minority traditional medicine. World Sci. Technology/Modernization Traditional Chin. Med. Materia Medica 21 (03), 449–456. Available online at: https://kns.cnki.net/kcms2/article/abstract?v=6RlcORkFSJTsahRmJLzjrToXuZ_UqLc4gweup3KEuhfVxBHY3PY2Vi01NWejYwTRxrX-vuw5wKd4KNhZNsEoHf7wyGOWDZJoDSZU-BQRmXTUjWWSNsMmQfLfeEBQKE5L_KhYIAnoKqmuuUeQb1eUXt6L8QQNodI3&uniplatform=NZKPT&language=CHS.

[B68] Liu Z. Gao W. Man S. Wang J. Li N. Yin S. (2012). Pharmacological evaluation of sedative-hypnotic activity and gastro-intestinal toxicity of Rhizoma Paridis saponins. J. Ethnopharmacol. 144 (1), 67–72. 10.1016/j.jep.2012.08.027 22960390

[B69] Liu Z. Gao W. Man S. Wang J. Li N. Yin S. (2012). Pharmacological evaluation of sedative–hypnotic activity and gastro-intestinal toxicity of Rhizoma Paridis saponins. J. Ethnopharmacol. 144 (1), 67–72. 10.1016/j.jep.2012.08.027 22960390

[B70] Lou J. Cui Y. Liu J. Zhao L. (2018). The physiological functions of phytosterols and their UnderlyingMechanism of regulating mitochondria. Prog. Biochem. Biophys. 45 (12), 1240–1249. 10.16476/j.pibb.2018.0116

[B71] Luo G. Luo T. Zhou Y. Zhou S. (1988). Preliminary study on the hemostatic effect of *paris* polyphylla saponin C. Pharmacol. Clin. Chin. Materia Medica (02), 37–40. Available online at: https://kns.cnki.net/kcms2/article/abstract?v=f1ZyUc11mdqEBbOo8RF1Ye3BYkb3WxChQyU1YL3VuyhsQqkHJ3uMsJPEoh7StXmDsXqVsYEOgST3GDHJYxFFcle-I8i8pYpPlNg7wm0G0MqW5-dnZoZ8DIvuhJBkBA_FseRY5X9VyXnz361vQ7YBAA==&uniplatform=NZKPT&language=CHS.

[B72] Luo Y. Sun Q. (2013). Guizhou ethnic common natural medicines volume 2. Guizhou Science and Technology Press. Available online at http://book.duxiu.com/bookDetail.jsp?dxNumber=000012427665&d=E00DDB715AADDEC38333796C1FF69040&fenlei=160518.

[B73] Ma K. (2016). Investigation record of achang ethnic medicine. Yunnan Ethnic Publishing House. Available online at http://book.duxiu.com/bookDetail.jsp?dxNumber=000016701891&d=7C7540123528AD7221DDFA732771256E&fenlei=160518.

[B74] Ma W. Ye J. (2013). Research and application of yunnan ethnic medicine in the prevention and treatment of skin diseases. Yunnan Science and Technology Press. http://book.duxiu.com/bookDetail.jsp?dxNumber=000015678724&d=E44121276A432C82C8B3B94C55139C3A&fenlei=160512.

[B75] Malla B. Gauchan D. P. Chhetri R. B. (2015). An ethnobotanical study of medicinal plants used by ethnic people in Parbat district of western Nepal. J. Ethnopharmacol. 165, 103–117. 10.1016/j.jep.2014.12.057 25571849

[B76] Man S. Gao W. Zhang Y. Huang L. Liu C. (2011). Identification of chemical constituents in Rhizoma Paridis Saponins and their oral administration in rat plasma by UPLC/Q-TOF/MS. Biomed. Chromatogr. 25 (6), 712–719. 10.1002/bmc.1507 20842760

[B77] Man S. Li J. Liu J. Chai H. Liu Z. Wang J. (2016). Curcumin alleviated the toxic reaction of Rhizoma Paridis saponins in a 45-day subchronic toxicological assessment of rats. Environ. Toxicol. 31 (12), 1935–1943. 10.1002/tox.22194 26390842

[B78] Medica, Medicine, Yunnan University Of Chinese (2009). Yunnan ethnic medicine chronicles volume 4. Yunnan Ethnic Publishing House. Available online at http://book.duxiu.com/bookDetail.jsp?dxNumber=000030268763&d=ABE2E84F04B87B49CA4584F41FC3FC79&fenlei=1605160103.

[B79] Mei X. (2018). Illustrated catalogue of Chinese She medicinal plants, 1. Zhejiang Science and Technology Press. Available online at http://book.duxiu.com/bookDetail.jsp?dxNumber=000018056292&d=45B70CFCC0B060BC41C5F1933CB190D9&fenlei=160518.

[B80] Meng W. Du J. Chen T. (2020). The chemical constituents and abtineoplastic effects of Paridis rhizoma. Chem. Life 40 (01), 70–74. 10.13488/j.smhx.20190114

[B81] Nie Q. Tan J. Chen Q. Kuang G. (2022). Advances in the study of the chemical composition and anti-tumor effects of Rhizoma Paridis saponins. Anti-tumor Pharm. 12 (03), 337–343. Available online at: https://link.cnki.net/urlid/43.1507.R.20220622.1127.002.

[B82] Niu Xu Li Z. Sun S. Jiang Ma Lin G. Shi W. (2020). Polyphyllin II inhibits human bladder cancer migration and invasion by regulating EMTassociated factors and MMPs. Oncol. Lett. 20, 2928–2936. 10.3892/ol.2020.11839 32782609 PMC7399771

[B83] Pan L. Haung R. Yan L. Zeng W. Lu Z. Daun W. (2023). Research progress on the regulation of insect resistance and biosynthesis by plant triterpenoid saponins. Jiangsu Agric. Sci. 51 (20), 1–8. 10.15889/j.issn.1002-1302.2023.20.001

[B84] Pan Q. Qin N. et al Committee, Zhuang Encyclopedia Compilation (1993). Zhuang encyclopedia dictionary. Guangxi People's Publishing House. Available online at: http://book.duxiu.com/bookDetail.jsp?dxNumber=000000404344&d=DA3B2282346962E93FC3E8F336C45CB7&fenlei=110307.

[B85] Panyadee P. Wittaya P. Tran Van O. N. Nghiem Duc T. Pham Thi Linh G. Le Thien K. (2024). Biodiversitas. Journal of Biological Diversity. Available online at: https://xueshu.baidu.com/usercenter/paper/show?paperid=1g5c0ap0nj5u0640r33c0re0h0267354&site=xueshu_se&hitarticle=1.

[B86] Paul A. Gajurel P. R. Das A. K. (2015). Threats and conservation of *Paris* polyphylla an endangered, highly exploited medicinal plant in the Indian Himalayan Region. Biodiversitas J. Biol. Divers. 16 (2), 295–302. 10.13057/biodiv/d160226

[B87] Pei S. (2007). Brief discussion on ethnomedicine research and new-drug development of China. J. Yunnan Coll. Traditional Chin. Medicine(04), 4–7. 10.19288/j.cnki.issn.1000-2723.2007.04.002

[B88] Peng Y. Zhang X. Li T. He J. (2019). Effects of extractive from *Paris* polyphylla on hemostasis for mice. J. Shaanxi Normal Univ. Nat. Sci. Ed. 47 (04), 97–101. 10.15983/j.cnki.jsnu.2019.04.344

[B89] Pfoze N. L. Kumar Y. Myrboh B. (2013). Screening of bioactive phytochemicals obtained from lesser known ethnomedicinal plants of Senapati district of Manipur, India.

[B90] Phurailatpam A. K. Choudhury A. Yatung T. Momin K. C. (2022). A review on the importance of two medicinal plants of North East India: *Paris* polyphylla Smith and Kaempheria parviflora Wall. ex Baker. Ann. Phytomedicine An Int. J. 11 (2). 10.54085/ap.2022.11.2.23

[B91] Qin X. Chen C. Ni W. Yan H. Liu H. (2013). C22-steroidal lactone glycosides from stems and leaves of *Paris* polyphylla var. yunnanensis. Fitoterapia 84, 248–251. 10.1016/j.fitote.2012.12.007 23262268

[B92] Qin X. Sun D. Ni W. Chen C. Hua Y. He L. (2012). Steroidal saponins with antimicrobial activity from stems and leaves of *Paris* polyphylla var. yunnanensis. Steroids 77 (12), 1242–1248. 10.1016/j.steroids.2012.07.007 22846376

[B93] Qin X. Yu M. Ni W. Yan H. Chen C. Cheng Y. (2016). Steroidal saponins from stems and leaves of *Paris* polyphylla var. yunnanensis. Phytochemistry 121, 20–29. 10.1016/j.phytochem.2015.10.008 26546502

[B94] Qin X. Zhang L. Zhang Y. Ni W. Yang X. Yu Q. (2020). Polyphyllosides A–F, six new spirostanol saponins from the stems and leaves of *Paris* polyphylla var. chinensis. Bioorg. Chem. 99, 103788. 10.1016/j.bioorg.2020.103788 32244126

[B95] Qin X. J. Ni W. Chen C. X. Liu H. Y. (2018a). Seeing the light: shifting from wild rhizomes to extraction of active ingredients from above-ground parts of *Paris* polyphylla var. yunnanensis. J. Ethnopharmacol. 224, 134–139. 10.1016/j.jep.2018.05.028 29792919

[B96] Qin X. J. Ni W. Chen C. X. Liu H. Y. (2018b). Seeing the light: shifting from wild rhizomes to extraction of active ingredients from above-ground parts of *Paris* polyphylla var. yunnanensis. J. Ethnopharmacol. 224, 134–139. 10.1016/j.jep.2018.05.028 29792919

[B97] Qiu W. Liu C. Ju Y. Zhang H. (2010). Pharmacokinetic interaction of plant preparations with chemical drugs. Chin. J. Nat. Med. 8 (02), 137–144. 10.3724/sp.j.1009.2010.00137

[B98] Sha A. Chen H. Zhao X. (2024). Exploration of the mechanisms of improving learning and memory in the offspring of aging pregnant mice by supplementation with *Paris* polyphylla polysaccharide based on the P19-P53-P21 and Wnt/β-catenin signaling pathways. J. Ethnopharmacol. 318 (Pt A), 116883. 10.1016/j.jep.2023.116883 37422103

[B99] Sharma A. Pallabi K. Hui T. (2015). Distribution and phytomedicinal aspects of Paris polyphylla Smith from the eastern himalayan region: a review. Tang 5, 15.1–15.12. 10.5667/tang.2015.0001

[B100] Shen F. Yang L. Peng Y. Tong X. Ma Y. (2010). Study on the antifertility effect of the saponins in rhizoma paridis yunnanensis [rhizoma from prais polyphylla Smith var. yunnanensis(franch) hand.-mazz.] *in vitro* . Chin. J. Mod. Appl. Pharm. 27 (11), 961–964. 10.13748/j.cnki.issn1007-7693.2010.11.004

[B101] Shen Y. Wang N. Gui D. (2021). Research progress of phytomedicine related acute kidney injury. World Clin. Drugs 42 (10), 819–828.

[B102] Shi G. Reng B. Du X. Zhang Q. Shi D. (2020). The effect of chicken excrement vine glycoside acid on IL-1 β - induced inflammatory response in articular chondrocytes. Chin. Tradit. Pat. Med. 42 (06), 1624–1627. Available online at: https://kns.cnki.net/kcms2/article/abstract?v=LY1OVaQjltyIBmjQ-4W-9dU9J0NtKxkyzRxB6-aWLCQuVzAWpKMv-lruoZHWuiBPxqsNpeiyeG8V7mGOrHPCA4Kta61sgUl_SMYVj8KSTkqhixo7COphNLXmyeBe3hOUiaZeR8n8Hm8ppINbfr6ToCutnfi025Awos0KCJVPrJ7aHDYrPAFH6Mhr2OhR8TpaZVf9ki2AmxQ=&uniplatform=NZKPT&language=CHS.

[B103] Su H. (2023). Study on the chemical constituents and fingerprint of the peels from *Paris* polyphylla. var. yunnanensis. Kunming Medical University, 96. 10.27202/d.cnki.gkmyc.2023.000311

[B104] Sun A. Wang Y. Hu G. Li L. Wang R. (2023). Polyphyllin I effects Candida albicans via inhibition of virulence factors. Evid.-based Complement. Altern. Med. 2023, 5645500–5645513. 10.1155/2023/5645500 PMC9886465 36726525

[B105] Tan L. Xiang M. Mi C. Li Z. Tian Z. Xiang B. (2017). The effect and mechanism of total saponins from *paris* polyphylla on airway inflammation in mouse asthma model. Chin. J. Gerontology 37 (19), 4703–4704. Available online at: https://kns.cnki.net/kcms2/article/abstract?v=bd42u7TqVAGb8LUeA2SySmoQVBiGRi00gsqSCx2LfmKETSpZp5x5oBLwriH10zd0jnZoiVUdBqDtGu94r0HPsnrblJ0ZcUqMsFCulfpf_KHGlo5SxlW4NXwWugnTxvfU_W3K8BI06Hnp5mkVWUM6HeQf7extt8Vt&uniplatform=NZKPT&language=CHS.

[B106] Tang N. Liu Y. Li Y. Lu Y. Shao Q. Yue Z. (2018). Chemical constituents from the root extract of *Paris* verticillata. Cent. South Pharm. 16 (3), 297–300. 10.7539/j.issn.1672-2981.2018.03.002

[B107] Tariq M. Paul S. Bhatt I. Chandrasekar K. Pande V. Nandi S. (2016). *PARIS* POLYPHYLLA SMITH: AN IMPORTANT HIGH VALUE HIMALAYAN MEDICINAL HERB. Int. J. Adv. Res. 4 (11), 850–857. 10.21474/ijar01/2157

[B108] Thakur U. Shashni S. Thakur N. Rana S. K. Singh A. (2023). A review on *Paris* polyphylla Smith: a vulnerable medicinal plant species of a global significance. J. Appl. Res. Med. Aromat. Plants. 33, 100447. 10.1016/j.jarmap.2022.100447

[B109] Tian Y. (2022). Studies on the steroidal saponins from *Paris* fargesii var. petiolata and the antitumor activity. Chinese People's Liberation Army Air Force Medical University, 131. 10.27002/d.cnki.gsjyu.2022.000228

[B110] Tian Y. Jia S. Shi J. Gong G. Yu J. Niu Y. (2019). Polyphyllin I induces apoptosis and autophagy via modulating JNK and mTOR pathways in human acute myeloid leukemia cells. Chem. Biol. Interact. 311, 108793. 10.1016/j.cbi.2019.108793 31421117

[B111] University T. F. M. M. (1976). Poisoning caused by Chinese herbal medicine and emergency treatment. Xian, 180. Available online at: http://book.duxiu.com/bookDetail.jsp?dxNumber=162036307356&d=DB011E428875D87243F1565353A1E1F7&fenlei=1608090504.

[B112] Wan Z. Zeng L. Zhou H. Li J. Lei C. (2022). Protective effect of polyphyllin Ⅶ on acute lung injury in rats with severe acute pancreatitis by inhibiting NF-κB signaling pathway. J. Jilin Univ. Med. Ed. 48 (03), 668–675. 10.13481/j.1671-587X.20220315

[B113] Wang C. W. Tai C. J. Choong C. Y. Lin Y. C. Lee B. H. Shi Y. C. (2016). Aqueous extract of *Paris* polyphylla (AEPP) inhibits ovarian cancer via suppression of peroxisome proliferator-activated receptor-gamma coactivator (PGC)-1alpha. Molecules 21 (6), 727. 10.3390/molecules21060727 27271583 PMC6273164

[B114] Wang S. Long S. Deng Z. Wu W. (2020). Positive role of Chinese herbal medicine in cancer immune regulation. Am. J. Chin. Med. 48 (7), 1577–1592. 10.1142/S0192415X20500780 33202152

[B115] Wang X. Chen Z. Yin H. (2018). “West China journal of pharmaceutical Sciences,” in The application of paris polyphylla in Chinese ethnic and folk medicine.

[B116] Wang Y. Fan Q. Xiang J. Huang H. Chen S. Liu B. (2020). Structural characterization and discrimination of *Paris* polyphylla var. yunnanensis by a molecular networking strategy coupled with ultra-high-performance liquid chromatography with quadrupole time-of-flight mass spectrometry. Rapid. Commun. Mass. Spectrom. 34 (11), e8760. 10.1002/rcm.8760 32065690

[B117] Wang Y. Gao W. Y. Zhang T. J. Guo Y. Q. (2007). A novel phenylpropanoid glycosides and a new derivation of phenolic glycoside from *Paris* Polyphylla var. yunnanensis. Chin. Chem. Lett. 18 (5), 548–550. 10.1016/j.cclet.2007.03.011

[B118] Wang Y. Jiang Y. Yang C. Wang J. Xu Z. Liu Y. (2022). Research progress on chemical constituents, pharmacological actities, and clinical applications of *Paris* polyphylla var. yunnanensis. Chin. Traditional Herb. Drugs 53 (23), 7633–7648. Available online at: https://kns.cnki.net/kcms2/article/abstract?v=bnAgTRNiloLI_GyZQ1UU7-oz1Q95cqbV6NMYc5aCPqECx0lpICh3YaqXODFbMtxlV3f-OdGOEIgkHBOoXfkU9Az9wwcNtHppobV4dD6Eoj46yvQrbTWzMP2_aieHDCS_rZw-YeHhI2TbmXdWAJ3TEH1h7tSSmdds&uniplatform=NZKPT&language=CHS.

[B119] Wang Y. Ma G. Li Y. Li Q. Gao Y. Yu X. (2024). Research progress on extraction and purification methods of phytosterols. CHINA OILS FATS 49 (02), 114–122.

[B120] Xiao N. Gai L. Luo B. Li H. Yang Z. (2016). Research progress on pharmacological effects of *paris* polyphylla. World Latest Medicne Inf. Electron. Version 16 (67), 51+50. Available online at: https://kns.cnki.net/kcms2/article/abstract?v=bnAgTRNiloIdWeR_YM7qdpKBUiZcGLXzNOhOFzv1yQLw_gOzFsUgoOTc0eOuSV8FVnomAd-ddqe4IDw19vhibAjmdzd2yRR2_7pJ7mKStxia9HIYfYYOkzj5C4ZcxjQYUO2i33PuXhfwv0ElGZKrfKfsES6_WlY3&uniplatform=NZKPT&language=CHS.

[B121] Xiao Y. Zhou X. Tang D. Zhang Y. Lin J. Chen T. (2021). Analgesic and anti-inflammatory eeffects of chonglou saponin Ⅶ, H and total saponins. J. Sichuan Traditional Chin. Med. 39 (06), 57–60. Available online at: https://kns.cnki.net/kcms2/article/abstract?v=f1ZyUc11mdq_xXfYd5LEjMaV9PABgBS0QOwsPZeaeQ2Gg6mtMKtjeJfUsccPr32MeP7FJ_dS6tSJkwR6st0jyQXNxlFzQDvMdFYJjqy79g1fNpPn_jZ689qrN75XIIlrnPx8BJvB6xMZ5uaPI1h2g_5qUXIxCjVl&uniplatform=NZKPT&language=CHS.

[B122] Xiong W. Xiu G. Zhou X. Yin Y. Ling B. Sun J. (2016). Research progress on clinical application of anti-inflammatory active ingredients in traditional Chinese medicine Rhizoma Paridis. Yunnan J. Traditional Chin. Med. Materia Medica 37 (09), 86–88. 10.16254/j.cnki.53-1120/r.2016.09.039

[B123] Xu H. Nie W. Lu L. Huang X. Wang H. Wan J. (2023). Study on chemical constituents of *Paris* polyphylla Smith var. Yunnanensis (Ⅰ). Guangzhou Chem. Ind. 51 (09), 63–65. Available online at: https://kns.cnki.net/kcms2/article/abstract?v=n93avYlexq8UNrHVAe8m0FNogxOLHg1Ck6wI1tlKJ30-VqPilWqk-U2K4dHULY5UM1pG2WHCvU0TiOF8UcpA0Y7VsP9-4kHlQgnIRQVOOEszQ2gLWlxZ81EC--q468SNXB8spDb9DIOu5HyGVKLg5QF56k9B4mga&uniplatform=NZKPT&language=CHS.

[B124] Xu X. Huang Y. (2017). Cultural chu xiong wuding. Yunnan People's Publishing House. Available online at http://book.duxiu.com/bookDetail.jsp?dxNumber=000017510815&d=7CE5D0ABF0773E510A17A903A7361809&fenlei=07020206.

[B125] Yang S. (2001). Jino ethnic medicine. Yunnan Science and Technology Press. Available online at http://book.duxiu.com/bookDetail.jsp?dxNumber=000001243986&d=5849F439044EA7D3F33815959E7D2357&fenlei=160518.

[B126] Yang W. Xiao B. Yu X. Han L. (2018). Research progress on reproductive toxicity of toxic traditional Chinesemateria medica recorded in Chinese Pharmacopoeia. Chin. J. Phamacol Toxicol. 32 (05), 355–363. Available online at: https://kns.cnki.net/kcms2/article/abstract?v=bnAgTRNiloL99iM49YIOXLoLbnY7QJTi0ti3fgXz7pfLKWwB7cCG-0fwPPLeUWBo5oTQ-kGYZUDovWbaVHSfwjG3XqUnQHOLGCZEofLYqL0LQsRiUK9SgiVYRLuJjcqQ9nVisYAz7EF-YtA1QiWh5zLf8kXYubiJ&uniplatform=NZKPT&language=CHS.

[B127] Yang Y. Bai X. Yang J. Luo J. Duan X. Wang Y. (2024). Studies on chemical constituents and antimicrobial activities of rhizomes of *Paris* tengchongensis. Chin. Traditional. Herb. Drugs 55 (05), 1466–1476. Available online at: https://kns.cnki.net/kcms2/article/abstract?v=6RlcORkFSJSPqwR_5M76M3sO6bpvpr4cTKli04FgLMzDW3ICzCTKFVVBkPyUn28eKkotPZWMb7f9nW09jjbmh6ej2Tgov1mZC5RpK4YmKfV5x3Ugg_9QM--HqglZ0twKU7kBPLL4b5SEbzXNVvneOFPIb397GnDX&uniplatform=NZKPT&language=CHS.

[B128] Yao N. Ren K. Wang Y. Jin Q. Lu X. Lu Y. (2017). *Paris* polyphylla suppresses proliferation and vasculogenic mimicry of human osteosarcoma cells and inhibits tumor growth *in vivo* . Am. J. Chin. Med. 45 (3), 575–598. 10.1142/S0192415X17500343 28385078

[B129] Ye X. Yang T. Xiu-Lan Pu Hong Hu Chen J. Lin T. C. (2025). The genus Paris: a fascinating resource for medicinal and botanical studies. Hortic. Res. 12, uhae327. 10.1093/hr/uhae327 40051578 PMC11883231

[B130] Yu H. Wu X. Zhang L. Tang H. (2012). Analysis of phytomedicines. Chin. J. Magnetic Reson. 29 (01), 128–141.

[B131] Yu Y. (2006). Chinese ethnic minority medicine and health care. China Intercontinental Press. Available online at http://find.nlc.cn/search/showDocDetails?docId=-9059521686489128024&dataSource=ucs01,fzdzsysj.

[B132] Yuan Y. L. Jiang N. Li Z. Y. Song Z. Z. Yang Z. H. Xue W. H. (2019). Polyphyllin VI induces apoptosis and autophagy in human osteosarcoma cells by modulation of ROS/JNK activation. Drug Des. Devel Ther. 13, 3091–3103. 10.2147/DDDT.S194961 PMC6717844 31695327

[B154] Zhang (2008). Complete book of folk secret formulas of various ethnic groups in China. Shanxi Science and Technology Press. Available online at: http://book.duxiu.com/bookDetail.jsp?dxNumber=000006640552&d=AFCB089B002CE37782335BFF08F79BF0&fenlei=16051705.

[B133] Zhang C. Li C. Jia X. Wang K. Tu Y. Wang R. (2019). *In vitro* and *in vivo* anti-inflammatory effects of polyphyllin VII through downregulating MAPK and NF-κB pathways. Molecules 24 (5), 875. 10.3390/molecules24050875 30832224 PMC6429153

[B134] Zhang C. Li Q. Qin G. Zhang Y. He C. Han L. (2021a). Anti-angiogenesis and anti-metastasis effects of Polyphyllin VII on Hepatocellular carcinoma cells *in vitro* and *in vivo* . Chin. Med. 16, 41. 10.1186/s13020-021-00447-w 34059099 PMC8166003

[B136] Zhang H. (2022). Study on chemical composition, preliminary activity and quality of extract from stems and leaves of the *Paris* polyphylla var. yunnanensis. Kunming Medical University, 87. 10.27202/d.cnki.gkmyc.2022.000122

[B137] Zhang H. Li Q. Gong L. Xu H. Su H. Feng L. (2023a). Chemical constituents of flavonoids from the stems and leaves of *Paris* polyphylla var. yunnanensis and their antiplatelet aggregation activities. Chin. Tradit. Pat. Med. 45 (03), 800–806. https://kns.cnki.net/kcms2/article/abstract?v=6RlcORkFSJTFkcGjbE9iEg3QSrcd2Vy-q2H_hZjxOcKpB_LaDuVwyxpGx4ufDrHRLvyV_GbHhpXHkw10rbjS41h9aXd7BPpbULKSYJ5kKelJCYKrvuaF_rzzI_4AGHC0DgSX5l9tEd47hLcK9lPOomBb56scvlrm&uniplatform=NZKPT&language=CHS.

[B138] Zhang X. Chen X. Liu M. Wang C. Zhou M. Zha Q. (2023b). Study on the distribution of medicinal plant resources of *Paris* and its application in ethnic minorities. Chin. Wild Plant Resour. 42 (01), 103–109+116. Available online at: https://kns.cnki.net/kcms2/article/abstract?v=f1ZyUc11mdra4MEJEsmchbbCYiBBKQWGnQ0vAnI2UWLAVG003bal_mdQTvbNTSqKIKCXmHGAznsu-XQwpP-jver99RnccT9ux2ylWez-DOW4uMJR74U1feWHfWGBANpvY9naVnwJguM02J9V1MiSQUnnP9LsZ_ec&uniplatform=NZKPT&language=CHS.

[B139] Zhang X. Liu Q. Liu B. (2021b). Advances in anti-tumor effects of paris saponins. Shaanxi J. Traditional Chin. Med. 42 (11), 1640–1643. 10.1186/s13020-021-00447-w

[B140] Zhang Y. Du J. (2021). Ethnopharmacology. Beijing: China Traditional Chinese Medicine Press, 310.

[B141] Zhang Y. Peng X. Wang C. Liu Y. Gao J. (2015). Research progress on active chemical components and pharmacological effects of *Paris* polyphylla Smith. Asia-Pacific Tradit. Med. 11 (02), 39–40. Available online at: https://link.cnki.net/urlid/42.1727.r.20150122.1404.015.

[B142] Zhang Y. Wu X. Li Y. Wang G. (2014). Chemical constituents of *Paris* polyphylla var. yunnanensis. J. Jinan Univ. Nat. Sci. & Med. Ed. 35 (01), 66–72. Available online at: https://link.cnki.net/urlid/44.1282.n.20140403.1106.021

[B143] Zhang Y. Zhang H. Fu J. Ruan Y. Yao A. Zhang P. (2024). Total saponins in paridis rhizoma: a review. Chin. J. Exp. Traditional Med. Formulae 30 (01), 232–243. 10.13422/j.cnki.syfjx.20232125

[B153] Zhang Zou (2017). Color Atlas of Miao Ethnic Medicine. Guizhou Science and Technology Press. Available online at http://book.duxiu.com/bookDetail.jsp?dxNumber=000030372997&d=BB28635612140BEA1BDD2C4108FF0FD8&fenlei=160518.

[B144] Zhao N. Yang Y. (2017). Color atlas of aquatic medicinal products. Guizhou Science and Technology Press. Available online at http://book.duxiu.com/bookDetail.jsp?dxNumber=000030357133&d=D3D2B2D6736677232E0EC3F563C0E138&fenlei=160518.

[B145] Zheng J. Chao Z. Qian Z. Medicine, Yunnan University Of Chinese (2019). Yunnan dictionary of ethnic medicine, 1. Shanghai Scientific and Technical Publishers. Available online at: https://kns.cnki.net/kcms2/article/abstract?v=n93avYlexq9DWqz1ugOwQj5w6bAvKNmneOSH4sHhKx24AzMiRzlKTTbHgHw4Y5OZBckb0OO1Gv7gAvyCn80Sk8pH41EzzpeiaCuxiyUKOz95Zqinbj28f7jAvf8weR2OkkBk4es0ztG8_HC0nmLZILtEq59F2J4m&uniplatform=NZKPT&language=CHS.

[B146] Zheng L. (2005). Studies on the synthesis and bioactivities of furostan saponin derivatives. Shenyang Pharmaceutical University, 82. Available online at https://kns.cnki.net/kcms2/article/abstract?v=n93avYlexq8utc2MgoTYXqdbUS83LrfXTTDVNKx0e9IPKObmMOnaz0HoTIKhutAMcg3AzHVWqP1obVj3kVtobv7eBBR7IaVzpCymR_uBjNiuAG6NLcBUEuO10jXjkZeRIW4vB2VcPFnt1VVEJ9aVaQh-5svDg-58&uniplatform=NZKPT&language=CHS.

[B147] Zheng Z. Tan X. Guan L. Wang R. Chen L. Wang Z. (2023). New steroidal saponins from aerial parts of *Paris* polyphylla var. chinensis. Zhongguo zhongyao zazhi 48 (17), 4589–4597. 10.19540/j.cnki.cjcmm.20230518.302 37802798

[B148] Zhong Y. Dan W. Xie J. Liu J. Shao Z. Yi F. (2019). Effects of total saponins from rhizoma *Paris* on radiosensitivity of hepatocellular carcinoma HepG_2 cells. Her. Med. 38 (06), 721–725.

[B149] Zhu L. Tan J. Wang B. Guan L. Liu Y. Zheng C. (2011). *In-vitro* antitumor activity and antifungal activity of pennogenin steroidal saponins from *paris* polyphylla var. yunnanensis. Iran. J. Pharm. Res. 10 (2), 279–286.Available online at: http://www.ncbi.nlm.nih.gov/entrez/query.fcgi?cmd=Retrieve&db=pubmed&dopt=Abstract&list_uids=24250355&query_hl=1. 24250355 PMC3828904

[B150] Zhu Y. Li X. Peng X. Tang R. Liu J. Ma W. (2024). Research progress of flavonoids in the field of plant protection. SHANDONG Chem. Ind. 53 (09), 110–113. Available online at: https://link.cnki.net/doi/10.19319/j.cnki.issn.1008-021x.2024.09.036.

[B151] Zhu Z. (2023). Exploration of the lnnovative development path of ethnic medicine. China Food & Drug Adm. Mag. 12, 12–17. Available online at: https://kns.cnki.net/kcms2/article/abstract?v=6RlcORkFSJSRD-qQHdKBlz_6Dq_tLNk6LeJQuRgAy9RZhaFkco5Lc3rCX4vath7r1fWjV5ku7-Gx15eqGfoHF1rnbbpU4zODqMBRJBcZc09vLwo3NcMwPgQNkBd-HXYXyWC3LEmbTYXYNt12JIg4Kkj6K59VtbTG&uniplatform=NZKPT&language=CHS.

[B152] Zuo W. Zhou N. Nong X. Sheng Y. (2015). Application of Winona medical skincare products in common skin diseases. J. Dermatology Venereol. 37 (2), 84–85. 10.3969/j.issn.1002-1310.2015.02.007

