# Supplementary material for: Paris spp (Liliaceae): a review of its botany, ethnopharmacology, phytochemistry, pharmacological activities, and practical applications
Source: Front Pharmacol. 2025 May 30;16:1570818. doi: 10.3389/fphar.2025.1570818 (PMC12162921; doi:10.3389/fphar.2025.1570818)
Supplement: Supplementary file 1 [file Table2.docx]

Supplementary Tables 2-13

# Supplementary Table 2

The structures of isosprirosterol saponins.

| Isosprirosterol | | | | | |
| --- | --- | --- | --- | --- | --- |
|          | | | | | |
|  | | | | | |
| No | R_1_ | R_2_ | R_3_ | R_4_ | R_5_ |
| 1 | Rha (1→2)-[ Ara (1→4)]-Glc | H | H | CH_3_ | H |
| 2 | Rha (1→2)-Glc | H | H | CH_3_ | H |
| 3 | Rha (1→2)-[Rha (1→4)-Rha (1→4)]-Glc | H | H | CH_3_ | H |
| 4 | Rha(1→2)-[Rha (1→4)]-Glc | H | H | CH_3_ | H |
| 5 | Glc | H | H | CH_3_ | H |
| 6 | Rha (1→3)-Glc | H | H | CH_3_ | H |
| 7 | Rha (1→2)-Rha (1→4)-[Rha (1→3)]-Glc | H | H | CH_3_ | H |
| 8 | Rha (1→4)[Rha (1→3)]-[Glc (1→2)]-Rha | H | H | CH_3_ | H |
| 9 | H | H | H | CH_3_ | H |
| 10 | Rha (1→2)-[Glc (1→3)]-Glc | H | H | CH_3_ | H |
| 11 | Ara (1→4)-Glc | H | H | CH_3_ | H |
| 12 | Rha (1→4)-Rha (1→4)-Glc | H | H | CH_3_ | H |
| 13 | Glc (1→2)-Glc | H | H | CH_3_ | H |
| 14 | Rha (1→4)-Glc | H | H | CH_3_ | H |
| 15 | Rha (1→2)-[Fuc (1→6)]-Glc | H | H | CH_3_ | H |
| 16 | Rha (1→2)-[Ara (1→4)]-[Fuc (1→6)]-Glc | H | H | CH_3_ | H |
| 17 | Rha (1→2)-[Rha (1→4)-Rha (1→4)]-Glc | H | H | CH_3_ | H |
| 18 | Rha (1→2)-[Api (1→3)]-Glc | H | H | CH_3_ | H |
| 19 | Rha (1→2)-[Ara (1→4)-Rha (1→5)]-Glc | H | H | CH_3_ | H |
| 20 | Rha (1→2)-[Ara (1→4)-Glc (1→5)]-Glc | H | H | CH_3_ | H |
| 21 | Glc (1→6)-Glc | H | H | CH_3_ | H |
| 22 | Rha (1→2)-[Glc (1→6)]-Glc | H | H | CH_3_ | H |
| 23 | Rha (1→2)-Rha (1→3)-Glc | H | H | CH_3_ | H |
| 24 | Rha (1→2)-[Ara (1→4)]-Glc | OH | H | CH_3_ | H |
| 25 | Rha (1→2)-Glc | OH | H | CH_3_ | H |
| 26 | Rha (1→2)-[Rha (1→4)]-Glc | OH | H | CH_3_ | H |
| 27 | Rha (1→2) [Rha (1→4)-Rha (1→4)]-Glc | OH | H | CH_3_ | H |
| 28 | Ara(1→4)-Glc | OH | H | CH_3_ | H |
| 29 | Rha(1→4)-Glc | OH | H | CH_3_ | H |
| 30 | Rha(1→2)-[Glc(1→3)]-Glc | OH | H | CH_3_ | H |
| 31 | Rha(1→2)-[Ara(1→4)]-Glc | OH | H | CH_3_ | H |
| 32 | Rha(1→2)-6-acetyl-Glc | OH | H | CH_3_ | H |
| 33 | Rha(1→4)-Rha(1→4)-Glc | OH | H | CH_3_ | H |
| 34 | Rha(1→2)-[Glc(1→5)-Ara(1→4)]-Glc | OH | H | CH_3_ | H |
| 35 | Rha(1→2)-[Xyl(1→5)-Ara(1→4)]-Glc | OH | H | CH_3_ | H |
| 36 | Rha(1→2)-[Api(1→4)]-Glc | OH | H | CH_3_ | H |
| 37 | Rha(1→2)-[Xyl(1→4)]-Glc | OH | H | CH_3_ | H |
| 38 | Glc | OH | H | CH_3_ | H |
| 39 | Rha(1→3)-Glc | OH | H | CH_3_ | H |
| 40 | Rha(1→2)-[Rha(1→3)]-Glc | OH | H | CH_3_ | H |
| 41 | Rha (1→2)-[Rha(1→5)-Ara (1→4)]-Glc | OH | H | CH_3_ | H |
| 42 | Glc(1→4)-Rha (1→2)-Glc | H | H | CH_3_ | H |
| 43 | Rha (1→2) [Glc(1→5)-Ara (1→4)]-Glc | H | H | CH_3_ | H |
| 44 | Rha (1→2) [Rha(1→5)-Ara (1→4)]-Glc | H | H | CH_3_ | H |
| 45 | Rha(1→2)-[Api(1→3)]-Glc | OH | H | CH_3_ | H |
| 46 | Xyl(1→5)-Ara(1→4)-Glc | OH | H | CH_3_ | H |
| 47 | Rha (1→2)-[Glc(1→4)-Rha (1→4)]-Glc | H | H | CH_3_ | H |
| 48 | Rha (1→2)-[Glc (1→4) ]-Glc | OH | H | CH_3_ | H |
| 49 | Rha (1→4)-[Rha (1→4)-Rha (1→2)]-Glc | H | H | CH_3_ | H |
| 50 | Rha (1→3)-[Ara (1→4)]-Glc | H | H | CH_3_ | H |
| 51 | Rha (1→4)-[Ara (1→2)]-Glc | H | H | CH_3_ | H |
| 52 | Rha (1→3))-[Rha (1→4)-Rha (1→2)]-Glc | OH | H | CH_3_ | H |
| 53 | H | OH | H | CH_3_ | H |

| Isosprirosterol | | | |
| --- | --- | --- | --- |
|   | | | |
| No | R | No | R |
| 54 | Rha(1→4)-Glc | 56 | Rha(1→2)-Glc |
| 55 | Rha(1→2)-Glc |  |  |

| Isosprirosterol | | | |
| --- | --- | --- | --- |
|  | | | |
| No | R_1_ | R_2_ | R_3_ |
| 57 | Rha(1→2)-[Ara(1→4)]-Glc | H | CH_3_ |
| 58 | Ara(1→4)-Glc | H | CH_3_ |
| 59 | Rha(1→2)-Glc | OH | CH_3_ |
| 60 | Rha(1→2)-[Rha(1→4)]-Glc | H | CH_3_ |
| 61 | Rha(1→2)-[Ara(1→4)]-Glc | OH | CH_3_ |
| 62 | Rha(1→2)-[Rha(1→4)-Rha(1→4)]-Glc | OH | CH_3_ |
| 63 | Rha(1→2)-[Glc(1→3)]-Glc | OH | CH_3_ |
| 64 | Rha(1→2)-[Glc(1→3)]-Glc | H | CH_3_ |
| 65 | H | H | CH_3_ |

| Isosprirosterol | | | |
| --- | --- | --- | --- |
|  | | | |
| No | R_1_ | R_2_ | R_3_ |
| 66 | Rha(1→2)-[Ara(1→4)]-Glc | OH | CH_3_ |
| 67 | Rha(1→2)-[Rha(1→4)]-Glc | OH | CH_3_ |
| 68 | Glc | H | CH_3_ |
| 69 | Rha(1→2)-Glc | H | CH_3_ |
| 70 | Rha(1→4)-Glc | H | CH_3_ |
| 71 | Rha(1→2)-[Rha(1→4)]-Glc | H | CH_3_ |
| 72 | Rha(1→2)-[Ara(1→4)]-Glc | H | CH_3_ |
| 73 | Rha(1→2)-[Rha(1→4)-Rha(1→4)]-Glc | OH | CH_3_ |
| 74 | Ara(1→4)-Glc | H | CH_3_ |
| 75 | H | H | CH_3_ |

| Isosprirosterol | | | |
| --- | --- | --- | --- |
|   | | | |
| No | R | No | R |
| 76 | Rha (1→3)-[Api (1→4)-Xyl (1→4)]-Ara | 81 | Rha (1→2)-[Rha (1→4)]-Glc |

| Isosprirosterol | | | |
| --- | --- | --- | --- |
|   | | | |
| No | R | No | R |
| 77 | Rha (1→2)-[Rha (1→4)-Rha (1→3)]-Glc | 80 | Rha (1→2)-[Glc (1→3)]-Glc |
| 78 | Rha (1→2)-[Ara (1→4)]-Glc |  |  |
| 79 | Rha (1→2)-[Rha (1→4)-Rha (1→4)]-Glc |  |  |

| Isosprirosterol | | | |
| --- | --- | --- | --- |
|   | | | |
| No | R | No | R |
| 82 | Rha (1→2)-[Rha (1→4)]-Glc | 83 | Rha (1→2)-[Rha (1→4)]-Glc |

| Isosprirosterol | | | |
| --- | --- | --- | --- |
|   | | | |
| No | R | No | R |
| 84 | Rha (1→2)-[Rha (1→4)]-Glc | 94 | Rha(1→2)-Glc |
| 85 | Rha (1→2)-[Rha (1→4)-Rha (1→4)]-Glc | 95 | Rha(1→2)-[Api(1→3)]-Glc |
| 86 | Rha (1→2)-[Rha (1→3)]-Glc |  |  |

| Isosprirosterol | | | |
| --- | --- | --- | --- |
|   | | | |
| No | R | No | R |
| 87 | Rha(1→2)-[Rha(1→4)]-Glc | 88 | Rha(1→2)-[Ara(1→4)]-Glc |

| Isosprirosterol | | | | |
| --- | --- | --- | --- | --- |
|   | | | | |
| No | R_1_ | R_2_ | No | R |
| 89 | Rha(1→2)-[Rha(1→4)-Rha(1→4)]-Glc | H | 96 | Rha(1→4)-Glc |
| 90 | Rha(1→2)-[Rha(1→4)-Rha(1→4)]-Glc | OH | 97 | Rha(1→2)-[Rha(1→4)]-Glc |
| 91 | Rha(1→2)-[Ara(1→4)]-Glc | H |  | |
| 92 | Rha(1→2)-[Rha(1→4)]-Glc | H |  | |
| 93 | Rha(1→2)-Glc | H |  | |

| Isosprirosterol | | | |
| --- | --- | --- | --- |
|   | | | |
| No | R | No | R |
| 98 | Rha(1→2)-[Rha(1→4)]-Glc | 99 | Rha(1→2)-[Rha(1→4)]-Glc |

# Supplementary Table 3

The structures of sprirostanol saponins.

| sprirostanol | | | | | |
| --- | --- | --- | --- | --- | --- |
|  | | | | | |
| No | R_1_ | R_2_ | R_3_ | R_4_ | R_5_ |
| 100 | Glc (1→6)-Glc | H | H | CH_2_OH | OH |
| 101 | Rha(1→2)-Rha(1→4)]-Glc | H | H | CH_2_OH | OH |
| 102 | Rha(1→2)-[Ara(1→4)]-Glc | OH | H | CH_2_OH | H |
| 103 | Rha(1→2)-[Ara(1→4)]-Glc | H | H | CH_2_OH | OH |
| 104 | Rha(1→2)-[Ara(1→4)]-Glc | OH | OH | CH_3_ | H |
| 105 | H | OH | H | CH_2_OH | H |
| 106 | Rha (1→2)-[Rha (1→4)-Rha (1→4)]-Glc | OH | H | CH_2_OH | OH |
| 107 | Rha (1→2) [Rha(1→4)-Rha (1→4)]-Glc | OH | H | CH_2_OH | H |
| 108 | Rha(1→2)-[Ara(1→4)]-Glc | OH | H | CH_2_O-Glc | H |
| 109 | Rha(1→4)-Rha (1→4)-Glc | OH | H | CH_2_O-Glc | H |
| 110 | Rha(1→2)-[Ara(1→4)]-Glc | OH | OH | CH_3_ | H |
| 111 | Rha(1→2)-[Ara(1→4)]-Glc | OH | O-Glc | CH_3_ | H |
| 112 | Rha (1→2)-[Rha (1→4) -Rha (1→4)]-Glc | OH | OH | CH_2_O-Glc | H |
| 113 | Rha (1→2)-[Rha (1→4) -Rha (1→4)]-Glc | OH | H | CH_2_O-Glc | H |
| 114 | Rha(1→2)-[Ara(1→4)]-Glc | OH | OH | CH_2_OH | H |
| 115 | Rha(1→2)-[Glc(1→3)]-Glc | OH | OH | CH_2_OH | H |
| 116 | Rha (1→2)-[Rha (1→4)]-Glc | OH | H | CH_2_OH | OH |
| 117 | Rha(1→2)-[Rha (1→4)]-Glc | OH | H | CH_2_OH | H |
| 118 | Rha (1→4)-Glc | OH | H | CH_2_OH | OH |
| 119 | Rha (1→4)-Glc | H | H | CH_2_OH | OH |
| 120 | Rha(1→2)-[Rha(1→4)]-Glc | H | H | CH_2_OH | O-Glc |
| 121 | Rha(1→2)-[Rha(1→4)]-Glc | H | H | CH_2_O-Glc | OH |
| 122 | RRha(1→2)-[Glc(1→3)]-Glc | H | H | CH_2_OH | OH |
| 123 | Rha (1→2)-[Rha (1→4) -Rha (1→4)]-Glc | H | H | CH_2_OH | H |
| 124 | Ara (1→4)-Glc | OH | H | CH_2_OH | H |
| 125 | Glc (1→6)-Glc | H | H | CH_2_OH | OH |
| 126 | Glc | H | H | CH_2_OH | O-Glc |
| 127 | Glc (1→6)-Glc | H | H | CH_2_OH | O-Glc |
| 128 | Rha (1→2)-Glc | OH | H | CH_2_OH | H |
| 129 | Rha(1→2)-[Ara(1→4)]-Glc | OH | H | CH_3_ | H |
| 130 | Rha(1→2)-[Glc(1→3)]-Glc | OH | H | CH_2_OH | H |
| 131 | Rha(1→2)-[Glc(1→4)-Rha(1→4)]-Glc | H | H | CH_2_O-Glc | H |
| 132 | Rha(1→2)-[Glc(1→3)]-Glc | H | H | CH_2_O-Glc | H |
| 133 | OH | OH | H | CH_2_OH | H |
| 134 | OH | OH | H | CH_2_OH | OH |
| 135 | Rha (1→2)-[Rha (1→4) -Rha (1→4)]-Glc | H | H | CH_2_O-Glc | H |

| sprirostanol | | | | | |
| --- | --- | --- | --- | --- | --- |
|   | | | | | |
| No | R_1_ | R_2_ | No | R_1_ | R_2_ |
| 136 | Xyl(1→4)-[Api(1→3)-Rha(1→2)]-Ara | H | 157 | Rha(1→2)-Glc | H |
| 137 | Rha(1→2)-[Xyl (1→3)]-Glc | Fuc | 158 | Rha(1→2)-[Api(1→3)]-Glc | H |
| 138 | Api (1→3)-Rha (1→2)-Xyl (1→3)-Ara | H | 159 | Rha(1→2)-[Ara(1→4)]-Glc | H |
| 139 | Rha(1→2)-[Xyl (1→3)]-Ara | H | 160 | Rha(1→2)-Glc | OH |
| 140 | Rha(1→2)-[Xyl (1→3)]-Glc | Fuc | 161 | Rha(1→2)-[Glc-(1→3)]-Glc | H |
| 141 | Xyl(1→3)-[Api(1→3)-Rha(1→2)]-Xyl | H | 162 | Rha(1→2)-[Rha(1→4)]-Glc | H |
| 142 | Rha(1→2)-[Xyl (1→3)]-Glc | Gal |  |  |  |
| 143 | Xyl(1→3)-[Api(1→3)-Rha(1→2)]-Glc | Fuc |  |  |  |

| sprirostanol | | | | | |
| --- | --- | --- | --- | --- | --- |
|   | | | | | |
| No | R_1_ | R_2_ | R_3_ | No | R |
| 144 | Api(1→3)-2,4-diacetyl-Rha(1→3)-Ara | CH_2_OH | H | 163 | Rha(1→2)-Glc |
| 145 | Api(1→3)-2-acetyl-Rha(1→4)-Ara | OCOCH_3_ | H | | |
| 146 | Xyl (1→3)-[Api(1→3)-Rha(1→2)]-Ara | CH_3_ | H | | |
| 147 | Xyl(1→3)-[Api(1→3)-Rha(1→2)]-Ara | CH_3_ | COCH_3_ | | |
| 148 | Xyl (1→4)-[Api(1→3)-Rha(1→2)]-Ara | CH_2_OH | H | | |
| 149 | Rha(1→2)-[Xyl (1→4)]-Ara | CH_3_ | COCH_3_ | | |

| sprirostanol | | | | | |
| --- | --- | --- | --- | --- | --- |
|   | | | | | |
| No | R_1_ | R_2_ | R_3_ | No | R |
| 150 | Rha(1→2)-Glc | OH | CH_2_OH | 155 | Rha(1→4)-Glc |
|  |  |  |  | 156 | Rha(1→2)-Glc |

| sprirostanol | | | | | |
| --- | --- | --- | --- | --- | --- |
|   | | | | | |
| No | R_1_ | R_2_ | No | R_1_ | R_2_ |
| 151 | Rha(1→2)-[Xyl(1→3)]-Glc | OH | 153 | Rha(1→2)-[Xyl-(1→3)]-Glc | O-Glc |
| 152 | Rha(1→2)-[Xyl(1→3)]-Glc | H | 154 | Rha(1→3)-[Api(1→4)-Xyl(1→4)]-Glc | H |

| sprirostanol | | | | |
| --- | --- | --- | --- | --- |
|   | | | | |
| No | R |  | No |  |
| 164 | Rha(1→4)-Glc |  | 165 |  |

| sprirostanol | |
| --- | --- |
|   | |
| No | No |
| 166 | 167 |

# Supplementary Table 4

The structures of cholestanol saponins.

| cholestanol saponins. | | | | |
| --- | --- | --- | --- | --- |
|   | | | | |
| No | R_1_ | R_2_ | No | R |
| 168 | Rha(1→2)-[Glc(1→3)]-Glc | Glc | 187 | Rha(1→2)-[Rha(1→4)]-Glc |
| 169 | Rha(1→2)-[Rha(1→4)-Rha(1→4)]-Glc | Glc |  |  |
| 170 | Rha(1→2)-Glc | Glc |  |  |
| 171 | Rha(1→2)-[Rha(1→4)]-Glc | Glc |  |  |
| 172 | Rha(1→2)-[Ara(1→4)]-Glc | Glc |  |  |
| 173 | Rha(1→2)-Glc | Ara(1→2)-Glc |  |  |

| cholestanol saponins. | | |
| --- | --- | --- |
|  | | |
| No | R_1_ | R_2_ |
| 174 | Rha(1→2)-[Ara(1→4)]-Glc | Glc |
| 175 | Rha(1→2)-[Ara(1→4)]-Glc | H |
| 176 | Rha(1→2)-Glc | H |
| 177 | Rha(1→2)-[Glc(1→3)]-Glc | H |
| 178 | Rha(1→2)-[Rha(1→4)-Rha(1→4)]-Glc | H |
| 179 | Rha(1→2)-[Glc(1→3)]-Glc | Glc |
| 180 | Rha(1→2)-[Rha(1→4)-Rha(1→4)]-Glc | Glc |
| 181 | Rha(1→2)-[Glc(1→5)-Ara(1→4)]-Glc | Glc |
| 182 | Rha(1→2)-[Xyl(1→5)-Ara(1→4)]-Glc | Glc |
| 183 | Rha(1→2)-[Ara(1→4)]-Glc | Glc |
| 184 | Rha(1→2)-[Ara(1→4)]-Glc | Glc(1→6)-Glc |
| 185 | Rha(1→2)-Glc | Ara(1→4)-Glc |
| 186 | Rha(1→4)-Rha(1→2)-Glc | Rha(1→4)-Glc |

| cholestanol saponins. | | | |
| --- | --- | --- | --- |
|   | | | |
| No | R | No | R_1_ |
| 188 | Rha(1→2)-[Rha(1→4)-Rha(1→4)]-Glc | 189 | Rha(1→2)-[Rha(1→4)-Rha(1→4)]-Glc |

| cholestanol saponins. | | |
| --- | --- | --- |
|  | | |
| No | R_1_ | R_2_ |
| 190 | Rha(1→2)-[Ara(1→4)]-Glc | Glc(1→6)-Glc |
| 191 | Rha(1→2)-[Rha(1→4)-Rha(1→4)]-Glc | Glc |

| cholestanol saponins. | | | | |
| --- | --- | --- | --- | --- |
|   | | | | |
| No | R_1_ | R_2_ | No | R |
| 192 | Rha(1→2)-[Rha(1→4)-Rha(1→4)]-Glc | H | 194 | Rha(1→2)-[Ara(1→4)]-Glc |
| 193 | Rha(1→2)-[Glc(1→3)]-Glc | Glc |  |  |

# Supplementary Table 5

The structures of furostanol saponins.

| furostanols | | | |
| --- | --- | --- | --- |
|   | | | |
| No | R_1_ | No | R |
| 195 | Rha(1→2)-Glc | 196 | Rha(1→2) |
|  |  | 197 | Glc(1→4)-[Rha(1→3)-Rha(1→3)]-Rha |
|  |  | 198 | Rha(1→2)-[Glc(1→3)]-Glc |
|  |  | 199 | Rha(1→2)-[Rha(1→4)-Rha(1→4)]-Glc |
|  |  | 200 | Rha(1→2)-[Ara(1→4)]-Glc |

| furostanols | | |
| --- | --- | --- |
|  | | |
| No | R_1_ | R_2_ |
| 201 | Rha(1→2)-[Rha(1→4)-Rha(1→4)]-Glc | OH |
| 202 | Rha(1→2)-[Glc(1→3)]-Glc | H |
| 203 | Glc(1→4)-[Rha(1→3)-Rha(1→3)]-Rha | H |
| 204 | Rha(1→2)-[Ara(1→4)]-Glc | H |
| 205 | Rha(1→2)-[Glc(1→3)]-Glc | OH |
| 206 | Rha(1→2)-[Glc(1→3)]-Glc | OH |
| 207 | Rha(1→2)-[Xyl(1→5)-Ara(1→4)]-Glc | OH |
| 208 | Rha(1→2)-[Rha(1→4)]-Glc | H |
| 209 | Rha(1→4)-Glc | H |
| 210 | Rha(1→2)-[Rha(1→4)]-Glc | OH |
| 211 | Rha(1→2)-Glc | OH |
| 212 | Rha(1→4)-Glc | OH |
| 213 | Rha(1→2)-[Glc(1→4)-Rha(1→4)]-Glc | H |
| 214 | Rha(1→2)-[Rha(1→4)-Rha(1→4)]-Glc | H |
| 215 | Rha(1→2)-[Ara(1→4)]-Glc | OH |
| 216 | Rha(1→3)-[Rha(1→4)]-Glc | H |

| furostanols | | | | |
| --- | --- | --- | --- | --- |
|  | | | | |
| No | R_1_ | R_2_ | R_3_ | R_4_ |
| 217 | Rha(1→2)-Glc | H | O-Glc | H |
| 218 | Rha(1→2)-[Ara(1→4)]-Glc | OH | O-Glc | H |
| 219 | Rha(1→2)-Glc | OH | O-Glc | H |
| 220 | Rha(1→2)-[Rha(1→4)]-Glc | OH | O-Glc | H |
| 221 | Rha(1→2)-[Rha(1→4)]-Glc | OCH_3_ | O-Glc | H |
| 222 | Rha(1→2)-[Rha(1→4)-Rha(1→4)]-Glc | OH | O-Glc | H |
| 223 | Rha(1→2)-[Glc(1→3)]-Glc | OCH_3_ | OH | H |
| 224 | Rha(1→2)-[Rha(1→4)-Rha(1→4)]-Glc | OH | O-Glc | H |

| furostanols | | | |
| --- | --- | --- | --- |
|  | | | |
| No | R_1_ | R_2_ | R_3_ |
| 225 | Rha-(1→4)-Rha-(1→2)-Glc | OH | Rha(1→4)-Glc |

| furostanols | | |
| --- | --- | --- |
|  | | |
| No | R_1_ | R_2_ |
| 226 | Rha(1→2)-[Rha(1→4)-Rha(1→4)]-Glc | H |
| 227 | Rha(1→4)-Rha(1→4)-Rha(1→4)-Glc | H |
| 228 | Rha(1→2)-[Rha(1→4)]-Glc | H |
| 229 | Rha(1→2)-Glc | H |
| 230 | Rha(1→4)-[Rha(1→4)-Rha(1→2)]-Glc | CH_3_ |
| 231 | Rha(1→2)-[Rha(1→4)]-Glc | CH_3_ |
| 232 | Glc(1→2)-[Glc(1→3)]-Glc(1→4)-Gal | CH_3_ |

| furostanols | | |
| --- | --- | --- |
|  | | |
| No | R_1_ | R_2_ |
| 233 | Rha(1→2)-[Rha(1→4)]-Glc | OH |

| furostanols | | |
| --- | --- | --- |
|  | | |
| No | R_1_ | R_2_ |
| 234 | Rha(1→2)-[Ara(1→4)]-Glc | Glc |

| furostanols | | | | |
| --- | --- | --- | --- | --- |
|   | | | | |
| No | R_1_ | R_2_ | No | R |
| 235 | Rha(1→3)-Rha(1→4)-Rha(1→4)-Glc | Glc | 242 | Rha(1→2)-[Rha(1→4)]-Glc |
| 236 | Rha(1→2)-[Rha(1→4)-Rha(1→4)]-Glc | Glc |  |  |
| 237 | Rha(1→2)-[Ara(1→4)]-Glc | Glc |  |  |
| 238 | Rha(1→2)-[Glc(1→3)-Glc(1→4)]-Glc | Glc |  |  |
| 239 | Rha(1→2)-[Glc(1→3)]-Glc | Glc |  |  |
| 240 | Rha(1→2)-Glc | Ara(1→4)-Glc |  |  |
| 241 | Rha(1→2)-Glc | Rha(1→4)-Glc |  |  |

| furostanols | | | | |
| --- | --- | --- | --- | --- |
|   | | | | |
| No | R | No | R_1_ | R_2_ |
| 243 | Ara(1→6)-Glc | 244 | Glc(1→4)-Gal | Glc |

| furostanols | | | | |
| --- | --- | --- | --- | --- |
|  | | | | |
| No | R | No | R_1_ | R_2_ |
| 245 | Rha(1→2)-[Rha(1→4)]-Glc | 246 | Rha(1→2)-[Rha(1→4)]-Glc | Glc |

# Supplementary Table 6

The structures of deformable sprirostanol saponins.

| deformable sprirostanols | | | |
| --- | --- | --- | --- |
|  | | | |
| No | R_1_ | R_2_ | R_3_ |
| 247 | Rha (1→2)-[Rha (1→4)]-Glc | OH | Glc |
| 248 | Rha (1→2)-[Rha (1→4)]-Glc | H | Glc |
| 249 | H | H | Glc |
| 250 | H | OH | Glc |
| 251 | Glc | H | Glc |
| 252 | Rha(1→2)-[Glc(1→3)]-Glc | H | Glc |
| 253 | Rha (1→2)-[Rha (1→4)]-Glc | OH | Glc |
| 254 | Rha (1→2)-[Rha (1→4)-Rha (1→4)]-Glc | H | Glc |
| 255 | Rha (1→2)-Glc | H | H |
| 256 | Rha (1→2)-Glc | H | Glc |
| 257 | Rha (1→4)-Glc | H | Glc |

| deformable sprirostanols | | | | | |
| --- | --- | --- | --- | --- | --- |
|   | | | | | |
| No | R_1_ | R_2_ | No | R_1_ | R_2_ |
| 258 | Rha (1→2)-Rha (1→4)-Glc | Glc | 259 | Rha(1→2)-Rha(1→4)-Glc | Glc |

| deformable sprirostanols | | |
| --- | --- | --- |
|  | | |
| No |  |  |
| 260 |  |  |

# Supplementary Table 7

The structures of other steroidal saponins.

| other steroidal saponins | | | |
| --- | --- | --- | --- |
|  | | | |
| No | R_1_ | R_2_ | R_3_ |
| 261 | Xyl(1→3)-[Api(1→3)-Rha(1→2)]-Glc | H | Fuc |
| 262 | Api(1→3)-[Api(1→3)-Rha(1→2)]-Glc | H | Fuc |
| 263 | Xyl(1→3)-[Rha(1→2)]-Glc | H | Fuc |
| 264 | Xyl(1→3)-[Rha(1→2)]-Glc | Gal | Fuc |

| other steroidal saponins | | | |
| --- | --- | --- | --- |
|  | | | |
| No | R_1_ | R_2_ | R_3_ |
| 265 | Xyl(1→3)-[Api(1→3)-Rha(1→2)]-Glc | Fuc | CH_3_ |
| 266 | Xyl(1→3)-[Api(1→3)-Rha(1→2)]-Glc | 6-decxy-Gul | CH_3_ |
| 267 | Xyl(1→3)-[Api(1→3)-Rha(1→2)]-Glc | 6-decxy-Gul | CH_2_OH |
| 268 | Xyl(1→3)-[Rha(1→2)]-Glc | 6-decxy-Gul | CH_2_OH |
| 269 | Xyl(1→3)-[Rha(1→2)]-Glc | Gal | O-Gal |
| 270 | Xyl(1→3)-[Api(1→3)-Rha(1→2)]-Glc | O-Fuc | Api |
| 271 | Rha(1→2)-[Xyl(1→6)-Glc(1→3)]-Glc | Gal | CH_3_ |
| 272 | Xyl(1→3)-[Rha(1→2)]-Glc | Gal | CH_2_OH |

# Supplementary Table 8

The structures of C-21 steroids

| C-21 steroids | | | |
| --- | --- | --- | --- |
|   | | | |
| No | R | No | R |
| 273 | Rha(1→2)-[Rha(1→4)]-Glc | 278 | Rha(1→2)-[Glc(1→3)]-Glc |
| 274 | Rha(1→2)-Glc | 279 | Rha(1→2)-[Ara(1→4)]-Glc |
| 275 | Rha(1→2)-[Ara(1→4)]-Glc | 280 | Rha(1→2)-[Rha(1→4)-Rha(1→4)]-Glc |

| C-21 steroids | | |
| --- | --- | --- |
|  | | |
| No | R_1_ | R_2_ |
| 276 | Rha(1→2)-[Xyl(1→3)]-Glc | Glc |
| 277 | Rha(1→2)-[Xyl(1→3)]-Glc | H |

| C-21 steroids | | | |
| --- | --- | --- | --- |
|  | | | |
| No | R_1_ | R_2_ | R_3_ |
| 281 | Rha(1→2)-[Rha(1→4)]-Glc | CH_3_ | OH |
| 282 | Rha(1→2)-[Rha(1→4)]-Glc | H | CH_3_ |

| C-21 steroids | | |
| --- | --- | --- |
|  | | |
| No | R_1_ | R_2_ |
| 283 | Rha(1→2)-[Rha(1→4)]-Glc | H |
| 284 | Rha(1→2)-Glc | H |
| 285 | Rha(1→2)-[Ara(1→4)]-Glc | H |
| 286 | Rha(1→2)-[Rha(1→4)-Rha(1→4)]-Glc | H |
| 287 | Rha(1→2)-[Rha(1→4)]-Glc | OCH_3_ |
| 288 | Rha(1→4)-[Rha(1→4)-Rha(1→2)]-Glc | H |

| C-21 steroids | | |
| --- | --- | --- |
|  | | |
| No | R_1_ | R_2_ |
| 289 | Rha(1→4)-Rha(1→4)-Rha(1→4)-Glc | Glc |

# Supplementary Table 9

The structures of phytosterols.

| phytosterols | | | |
| --- | --- | --- | --- |
|   | | | |
| No | R | No | R |
| 290 | Glc(1→2)-[Glc(1→6)]-Glc | 293 | Glc(1→2)-[Glc(1→6)]-Glc |
| 291 | Glc | 294 | Glc |
| 292 | H | 295 | H |

| phytosterols | | | |
| --- | --- | --- | --- |
|   | | | |
|  | No | No | R |
|  | 296 | 297 | Glc |

| phytosterols | | |
| --- | --- | --- |
|   | | |
| No | R | No |
| 298 | Glc | 299 |

# Supplementary Table 10

The structures of insect allergy hormones.

| insect allergy hormones | | | | | |
| --- | --- | --- | --- | --- | --- |
|  | | | | | |
| No | R_1_ | R_2_ | R_3_ | R_4_ | R_5_ |
| 300 | H | OH | OH | H | H |
| 301 | H | OH | OH | OH | OH |
| 302 | OH | H | OH | H | H |
| 303 | OH | OH | OH | H | H |
| 304 | H | OH | OH | OH | H |

| insect allergy hormones | | | |
| --- | --- | --- | --- |
|   | | | |
| No | 305 | No | 306 |

| insect allergy hormones | | | |
| --- | --- | --- | --- |
|   | | | |
| No | 307 | No | 308 |

| insect allergy hormones | |
| --- | --- |
|  | |
| No | 309 |

# Supplementary Table 11

The structures of triterpenes.

| triterpenes | | |
| --- | --- | --- |
|  S1: S2 :  | | |
| No | R_1_ | R_2_ |
| 310 | Rha(1→4)-Ara(1→2)-Xyl | Glc |
| 311 | Rha(1→2)-Ara | Rha(1→6)-Glc(1→6)-Glc(1→2)-Ara(1→4)-Rha(1→4)-Glc |
| 312 | 6′-methyl ester-Glc | Glc |
| 313 | 6′-butyl ester-Glc | Glc |
| 314 | Glc(1→2)-Ara | H |
| 315 | Glc(1→2)-Xyl | H |
| 316 | Ara | H |
| 317 | Xyl | H |
| 318 | glucuronide | H |
| 319 | Rha(1→2)-Glc | H |
| 320 | Glc(1→2)-Glc | H |
| 321 | Glc(1→2)-Ara | Rha(1→4)-Glc(1→6)-Glc |
| 322 | Glc(1→3)-Ara | Rha(1→4)-Glc(1→6)-Glc |
| 323 | Rha(1→4)-Ara | Rha(1→6)-Glc(1→6)-Glc(1→2)-Ara(1→4)-Rha(1→4)-Glc |
| 324 | Rha(1→2)-Ara | H |
| 325 | S1 | Rha(1→4)-Glc(1→6)-Glc |
| 326 | S2 | Rha(1→4)-Glc(1→6)-Glc |
| 327 | H | Rha(1→4)-Glc(1→6)-Glc |
| 328 | H | H |

| triterpenes | | | |
| --- | --- | --- | --- |
|   | | | |
| No | 329 | No | 330 |

| triterpenes | | | |
| --- | --- | --- | --- |
|   | | | |
| No | 331 | No | 332 |

| triterpenes | | | |
| --- | --- | --- | --- |
|   | | | |
| No | R | No | R |
| 333 | Glc(1→2)-Ara | 335 | Glc(1→2)-Ara |
| 334 | Glc(1→2)-Xyl | 336 | Glc(1→2)-Xyl |

| triterpenes | | | |
| --- | --- | --- | --- |
|   | | | |
| No | R | No | R |
| 337 | Glc(1→2)-Ara | 339 | Xyl(1→2)-Ara |
| 338 | Glc(1→2)-Xyl | 340 | Glc(1→4)-Ara |
|  |  | 341 | Rha(1→2)-Ara |
|  |  | 342 | Xyl |

| triterpenes | | | |
| --- | --- | --- | --- |
|   | | | |
| No | R | No | R |
| 343 | Rha(1→2)-Ara | 344 | H |
|  |  | 345 | CO(CH2)12CH3 |

# Supplementary Table 12

The structures of flavonoids.

| flavonoids | | | | | | |
| --- | --- | --- | --- | --- | --- | --- |
|  | | | | | | |
| No | R_1_ | R_2_ | R_3_ | R_4_ | R_5_ | R_6_ |
| 346 | Glc(1→6)-Glc | OH | OH | OCH_3_ | H | OH |
| 347 | Rha(1→2)-Glc | OH | OH | OH | H | OCH_3_ |
| 348 | Glc | OH | O-Rha | OCH_3_ | H | OH |
| 349 | Glc | OH | OH | H | OCH_3_ | OH |
| 350 | H | OH | OH | H | H | OH |
| 351 | H | OH | OH | H | OCH_3_ | OH |
| 352 | Glc | OH | OH | H | OH | OH |
| 353 | Rha(1→6)-Gal | OH | OH | H | H | OH |
| 354 | Rha(1→6)-Glc | OH | OH | H | H | OH |
| 355 | Rha(1→6)-Glc | OH | OH | OCH_3_ | H | OH |
| 356 | Gal(1→6)-Gcl | OH | OH | OCH_3_ | H | OH |
| 357 | Glc(1→2)-Gal | OH | OH | H | H | OH |
| 358 | Gal | OH | OH | H | H | OH |
| 359 | Glc | OH | OH | H | OCH_3_ | OH |
| 360 | Glc | OH | OH | H | H | OH |
| 361 | Glc(1→2)-Gal | OH | OH | H | OCH_3_ | OH |
| 362 | Glc(1→2)-Glc | OH | OH | H | H | OH |
| 363 | H | O-Rha | OH | H | H | OH |
| 364 | Glc | OH | O-Rha | H | H | OH |
| 365 | Glc(1→6)-Glc | OH | O-Glc | H | H | OH |
| 366 | Gal(1→6)-Glc | OH | OH | H | H | OH |
| 367 | H | OH | OH | H | OH | OH |
| 368 | Glc | OH | OH | H | OH | OH |
| 369 | Gal | OH | OH | OH | H | OH |
| 370 | H | OH | Glc | H | H | OH |
| 371 | Glc(1→3)-Glc | OH | O-Rha | H | H | OH |
| 372 | Ara(1→6)-Gal | OH | OH | H | H | OH |
| 373 | Rha(1→6)-Glc | OH | OH | OH | H | OH |
| 374 | Glc | OH | OH | OH | OH | OH |

| flavonoids | |
| --- | --- |
|  | |
| No 375 | No 376 |

| flavonoids | |
| --- | --- |
|  | |
| No 377 | No 378 |

| flavonoids | |
| --- | --- |
|  | |
| No | 379 |

# Supplementary Table 13

The structures of other chemical compounds

| others | | | | | |
| --- | --- | --- | --- | --- | --- |
|  | | | | | |
| No | R_1_ | R_2_ | R_3_ | R_4_ | R_5_ |
| 380 | OCH_3_ | O-Glc | O-Glc | OCH_3_ | OCH_3_ |
| 381 | OCH_3_ | O-Glc | O-Glc | H | H |
| 382 | OCH_3_ | O-Glc | O-Glc | H | OCH_3_ |

| others | | | | |
| --- | --- | --- | --- | --- |
|  | | | | |
| No | R_1_ | R_2_ | R_3_ | R_4_ |
| 383 | OH | H | Glc | H |
| 384 | O-Glc | H | H | H |
| 385 | OH | OCH_3_ | Glc | OCH_3_ |

| others | | |
| --- | --- | --- |
|  | | |
| No | R | No |
| 386 | Glc | 387 |

| others | |
| --- | --- |
|  | |
| 388 | 389 |

| others |
| --- |
| 390 R=Glc(1→6)-Glc |
|  |
| 391 R=Glc(1→5)-Glc 392 |
| 393 394 |
| 395 |
| 396 397 |
| 398 399 |
| 400 401 |
| 402 403 |
| 404 |
| 405 |
| 406 407 |
| 408 409 |
| 410 411 |
| 412 |
| 413 414 |
| 415 416 |
| 419 R=Glc 420 R1=CH3 |
| 421 422 |
| 423 425 |
| 424 426 |
| 427 428 429 |
| 430 431 R=Glc(→6Glc) |
